# Neutrophil degranulation and extracellular ROS production are inactivated by *Yersinia pseudotuberculosis* YopE through a SKAP2 independent pathway

**DOI:** 10.1128/mbio.01984-26

**Published:** 2026-10-06

**Authors:** Pathricia Angel Leus, Claudia Mañan Mejias, Alison Ren, Majorie de la Rosa, Emma Lofgren, Steven C. Bunnell, David B. Sykes, Joan Mecsas

**Affiliations:** 1 Graduate Program in Molecular Microbiology, Tufts Graduate School of Biomedical Sciences 50910, Boston, Massachusetts, USA; 2 Department of Molecular Biology and Microbiology, Tufts University School of Medicine 12261, Boston, Massachusetts, USA; 3 Department of Immunology, Tufts University School of Medicine 12261, Boston, Massachusetts, USA; 4 Center for Regenerative Medicine, Massachusetts General Hospital 2348, Boston, Massachusetts, USA; University of California, Berkeley, Berkeley, California, USA

**Keywords:** neutrophils, Yersinia, Yop, degranulation, HoxB8, RhoG, SKAP2

## Abstract

Upon sensing *Yersinia pseudotuberculosis* (*Yptb*), receptor-mediated pathways are stimulated to trigger polymorphonuclear (PMN) antimicrobial responses. *Yptb* injects multiple type 3 secreted effector proteins, Yops (*Yersinia* outer proteins), that possess distinct biochemical functions, into PMNs to inhibit PMN responses. We show that several Yops, YopE, YopH, and YopO, each partially interfered with CD63 plasma membrane localization, a marker for primary degranulation. The host pathways involved in CD63 mobilization are complex, and it is not completely understood how Yops collaborate to inactivate this process. Here, CRISPR/Cas9 technology was used in an immortalized system of myeloid progenitor cells (Cas9-ER-HoxB8) to generate knockout PMN cell lines. To probe the impact of different Yops on the neutrophil pathways activated upon encountering *Yptb,* we interrogated the panel of genetically modified neutrophils with genetically modified bacteria. This approach of targeted gene deletion to inactivate specific pathways/proteins uncovered host pathways that synergize to induce CD63 mobilization that are distinctly targeted by YopE and YopH. YopE inhibited CD63 mobilization in the absence of SKAP2, a YopH target, whereas YopH inhibited CD63 mobilization in the absence of RhoG, a YopE target, indicating that these Yops inactivate distinct signaling pathways. Furthermore, the SKAP2-independent pathway inactivated by YopE is involved in primary granule release and ROS production. Overall, this work highlights multiple signaling pathways involved in mounting degranulation and ROS production and the diverse Yop-mediated mechanisms that WT-*Yptb* employs to effectively disarm them. Importantly, this work provides an avenue to untangle neutrophil signaling pathways targeted by pathogens using Cas9-ER-HoxB8 cells.

**IMPORTANCE:**

When sensing invading bacteria, neutrophils become activated through multiple receptors that trigger signal-transduction cascades resulting in the generation of antimicrobial responses. The enteric pathogen, *Yersinia pseudotuberculosis* (*Yptb*), is equipped to effectively inhibit these responses using its collection of effector proteins (Yops). Here, we developed a system to overcome the limitations of performing genetic manipulations in neutrophils by implementing CRISPR/Cas9 technology in an engineered system of myeloid progenitor cells (Cas9-ER-HoxB8), which can be induced to differentiate into neutrophils. By infecting genetically modified neutrophils with *Yptb* strains expressing individual Yops, we identified distinct host signaling pathways that synergize to induce neutrophil antimicrobial responses. Our findings provide insight into several signaling events triggered by *Yptb* infection and show how YopE and YopH target distinct pathways to block vesicle trafficking and extracellular ROS production. This powerful genetic system can be applied to other pathogens to dissect the intricacies of neutrophil-pathogen interactions.

## INTRODUCTION

Upon sensing the Gram-negative bacterium *Yersinia pseudotuberculosis* (*Yptb*), various neutrophil cell surface receptors are stimulated, including integrins, Toll-like receptors (TLR), and G-protein-coupled receptors (GPCRs) (1–4). Activation of these receptors induces signaling cascades that trigger neutrophil effector responses including degranulation, extracellular vesicle (EV) release, phagocytosis, and reactive oxygen species (ROS) production. To counter these host defenses, the three human pathogenic *Yersinia* species, *Yptb, Y. enterocolitica,* and *Y. pestis,* inject 6–7 effector proteins, called Yops, into neutrophils via a type 3 secretion system (T3SS) (5, 6). Neutrophils are the primary cell targets for Yop translocation during murine infection (7–9). Collectively, Yops effectively inhibit many neutrophil effector responses, including degranulation (4, 7–12).

Neutrophils harbor different subsets of granules, primary, secondary, tertiary, and secretory, that are released in a sequential fashion upon sensing a pathogen in a process called degranulation. Primary granules are the last subset of granules released by neutrophils and contain the most potent antimicrobial molecules, including myeloperoxidase (MPO) and elastase. After *Y. pestis* infection, a combination of several Yops, YopE, YopH, or YopO (YpkA), coordinates to block CD63 plasma membrane localization, a marker for primary degranulation (11, 12). In the context of *Yptb* infection, deletion of both YopE and YopH (∆*yopEH*) abolishes *Yptb*’s ability to inhibit primary degranulation as measured by release of MPO, suggesting that one or both of these Yops are required to inhibit primary degranulation (10). The requirement for multiple Yops to inhibit primary degranulation suggests that multiple pathways must be inactivated to completely block this process. This is supported by inhibitor studies with *Y. pestis,* which implicate both Ca^2+^ influx and Rac activity as the key processes targeted by YopE and YopH to inhibit primary granule mobilization (11). However, the receptors and subsequent signaling events stimulated by the enteric *Yersinia* pathogens are distinct from those triggered by *Y. pestis*, with the enteric *Yptb* triggering integrin receptors, CCR5, and TLR-4, while *Y. pestis* triggers FPR1 (1–3, 13).

The biochemical activities of the Yops are conserved among the three *Yersinia* human pathogens. YopH is a tyrosine phosphatase, and various proteins involved in integrin-mediated neutrophil signaling, including Src kinase-associated phosphoprotein 2 (SKAP2), SH2 domain-containing leukocyte protein of 76 kDa (SLP-76), Vav guanine nucleotide exchange factor 1 (VAV1), and phospholipase C gamma 2 (PLCγ2), are dephosphorylated in the presence of YopH (4, 14–17). Of note, the YopH target, SKAP2, is a key adaptor protein in neutrophil signaling and is required for maximal ROS production but not degranulation (4, 14). Furthermore, growth of a ∆*yopH* mutant is partially restored in a *Skap2^−/−^* mice, indicating that SKAP2 is one but not the only critical target of YopH, and therefore, other antimicrobial processes, such as degranulation, are involved in controlling the growth of ∆*yopH* (4). YopE is a GTPase-activating protein (GAP) and inactivates the small Rho GTPases, Rac1, Rac2, RhoA, and RhoG (18–22). The YopE targets, Rac2 and RhoA, have both been implicated in neutrophil primary degranulation because of their involvement in actin cytoskeletal rearrangements. Specifically, activated Rac2 is essential for CD63 plasma membrane localization and actin polymerization (23–25), while inactivation of RhoA around the granules leads to blockage of actin polymerization, and thus allows for trafficking of the granule to its target membrane (26, 27). To our knowledge before this work, RhoG had not yet been implicated in degranulation.

In this work, we developed a genetic system in neutrophils to identify specific proteins and signaling pathways targeted by different *Yptb* Yops to inhibit primary degranulation. Prior to this work, the ability to perform experiments with neutrophils containing targeted genetic deletions was limited. Previous approaches involve the use of bone marrow-derived neutrophils from knockout (KO) or conditional KO mice and/or chemical inhibitors to inactivate the function of targeted proteins (4, 10, 11). However, chemical inhibitors can have toxic, pleiotropic, and off-target effects, and PMNs isolated from KO mice can be limiting since targeting of some genes results in lethality, including *Rac1*, *RhoA*, and *Syk* (28–30). While tissue conditional KOs have become more available, these are still limited and can be costly (31, 32).

To bypass these limitations and evaluate the roles of specific PMN genes, we generated a Cas9-expressing myeloid progenitor cell line using the conditional expression of HoxB8 under the control of estrogen (the ER-HoxB8 system) (33, 34). This permitted targeted genetic deletions of many of the known and hypothesized Yop targets in immortalized ER-HoxB8 myeloid progenitor cells, which we then differentiated into PMNs (KO-PMNs). To examine the contribution of each Yop, we paired genetically modified host and genetically modified bacteria, where the newly generated KO-PMNs were infected with a panel of *Yersinia* strains expressing individual Yops (Yop-only). We hypothesized that inactivation of both a Yop-targeted pathway using a Yop-only strain and another pathway through the deletion of the PMN gene would delineate distinct pathways targeted by individual Yops. Using this strategy, we uncovered that YopE acts independently of the YopH-targeted pathway, where YopE more effectively blocked degranulation and extracellular ROS production in the absence of the YopH target, SKAP2. Additionally, YopE’s impact on CD63 mobilization was enhanced in the absence of SLP-76 and PLCγ2, which are two other proteins dephosphorylated in the presence of YopH. Furthermore, our findings demonstrated that YopH acts on the RhoG-independent pathway to halt CD63 plasma membrane localization, overall supporting the idea that YopE and YopH target distinct signaling pathways.

## RESULTS

### Creation of neutrophil knockout cell lines using a Cas9-GFP-ER-HoxB8 myeloid progenitor cell line

**Fig 1:**
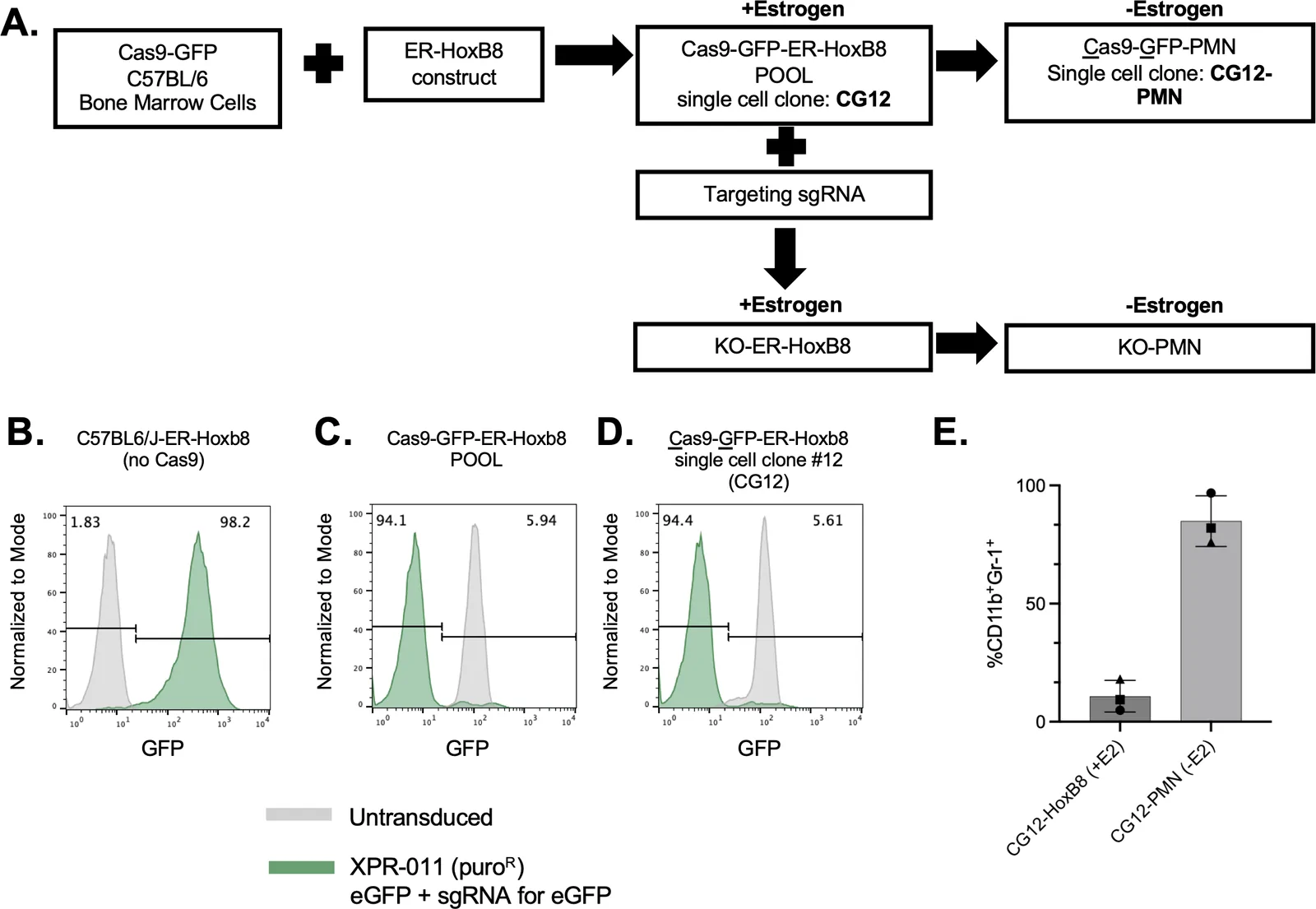
Cas9-ER-Hoxb8 cells have high Cas9 efficiency. (**A**) Schematic for generating knockout PMN (KO-PMN) cell lines. Myeloid progenitor cells isolated from bone marrow of a Cas9-GFP mouse were transduced with a construct encoding for an estrogen-regulated expression of HoxB8 to generate a pool of Cas9-GFP-expressing myeloid progenitor HoxB8 cells (Cas9-GFP-HoxB8s). Cas9-GFP-ER-HoxB8 single-cell clone, CG12, was transduced with sgRNAs to generate KO-ER-HoxB8 cells, which can be differentiated into KO-PMNs after removal of estrogen. (**B through D**) Histogram of (**B**) C57BL/6J-ER-HoxB8, (**C**) Cas9-GFP-ER-HoxB8 Pool, or (**D**) CG12, after transduction with pXPR-011 and 7 days of selection with 2 µg/mL puromycin. Cells were analyzed on BD LSRII for GFP expression (green peak) and compared to their respective untransduced cell line (gray peak). (**E**) CG12 was grown in the presence of estrogen (+E2) or was differentiated into CG12-PMN’s (−E2) (see Materials and Methods) for 3 days. CD11b and Gr-1 expression were assessed by flow cytometry. Each dot represents a biological replicate, which are matched by symbol. Bars are mean ± SD of three biological replicates.

To uncover distinct neutrophil pathways impacted by different Yops, we performed a dual-genetics screen and infected a panel of neutrophil knockout cell lines (KO-PMNs) with various *Yptb* Yop mutants, each expressing an individual Yop. This first required the generation of neutrophil KO cell lines lacking genes that encode for known/hypothesized *yop* targets. Because neutrophils are difficult to genetically manipulate, we implemented CRISPR/Cas9 in ER-HoxB8-transduced myeloid progenitor cells (33, 34). To make targeted deletions in myeloid progenitor cells, we harvested bone marrow cells from transgenic Cas9-GFP-expressing C57BL/6J mice and immortalized them through transduction with an estrogen-regulated (ER) HoxB8 construct to generate Cas9-GFP-ER-HoxB8 (Fig. 1A) (34).

To assess the efficiency of Cas9 gene editing, we transduced the reporter construct, pXPR-011, into Cas9-GFP-ER-HoxB8s (35). This construct contains (i) the gene encoding eGFP, (ii) an sgRNA for eGFP, and (iii) a puromycin-resistance cassette. In the presence of active Cas9 in the Cas9-GFP-ER-HoxB8 cells, the sgRNA for eGFP will direct the Cas9 endonuclease to induce a double-stranded break in the GFP gene in both the reporter construct and the Cas9 transgene, resulting in loss of GFP expression. Thus, the absence of GFP expression is a proxy measure for Cas9 efficiency. ER-HoxB8 cells generated from WT C57BL/6J mice (WT-ER-HoxB8), which lack the Cas9-GFP transgene and GFP, were used as a control and were successfully transduced, with 98.2% of the cells expressing GFP after puromycin selection (Fig. 1B). By contrast, 94.1% of the Cas9-GFP-ER-HoxB8 pool lacked GFP expression after transduction of pXPR-011, indicating that the Cas9 efficiently cleaved the genes for the reporter GFP and the Cas9-GFP in the cells (Fig. 1C). CG12, a single-cell clone derived from the Cas9-GFP-ER-HoxB8 pool, also exhibited robust Cas9 activity with 94.4% of cells losing GFP expression (Fig. 1D). This clone differentiated efficiently into CG12-PMN after removal of estrogen in the presence of IL-3 and GCSF (granulocyte colony-stimulating factor), as demonstrated by increased expression of CD11b and Gr-1 (Fig. S1B and Fig. 1E) and was used as the parental strain for generating KO cell lines (see below).

### YopH, YopE, and YopO play roles in reducing CD63 plasma membrane localization after *Yptb* infection

**Fig 2:**
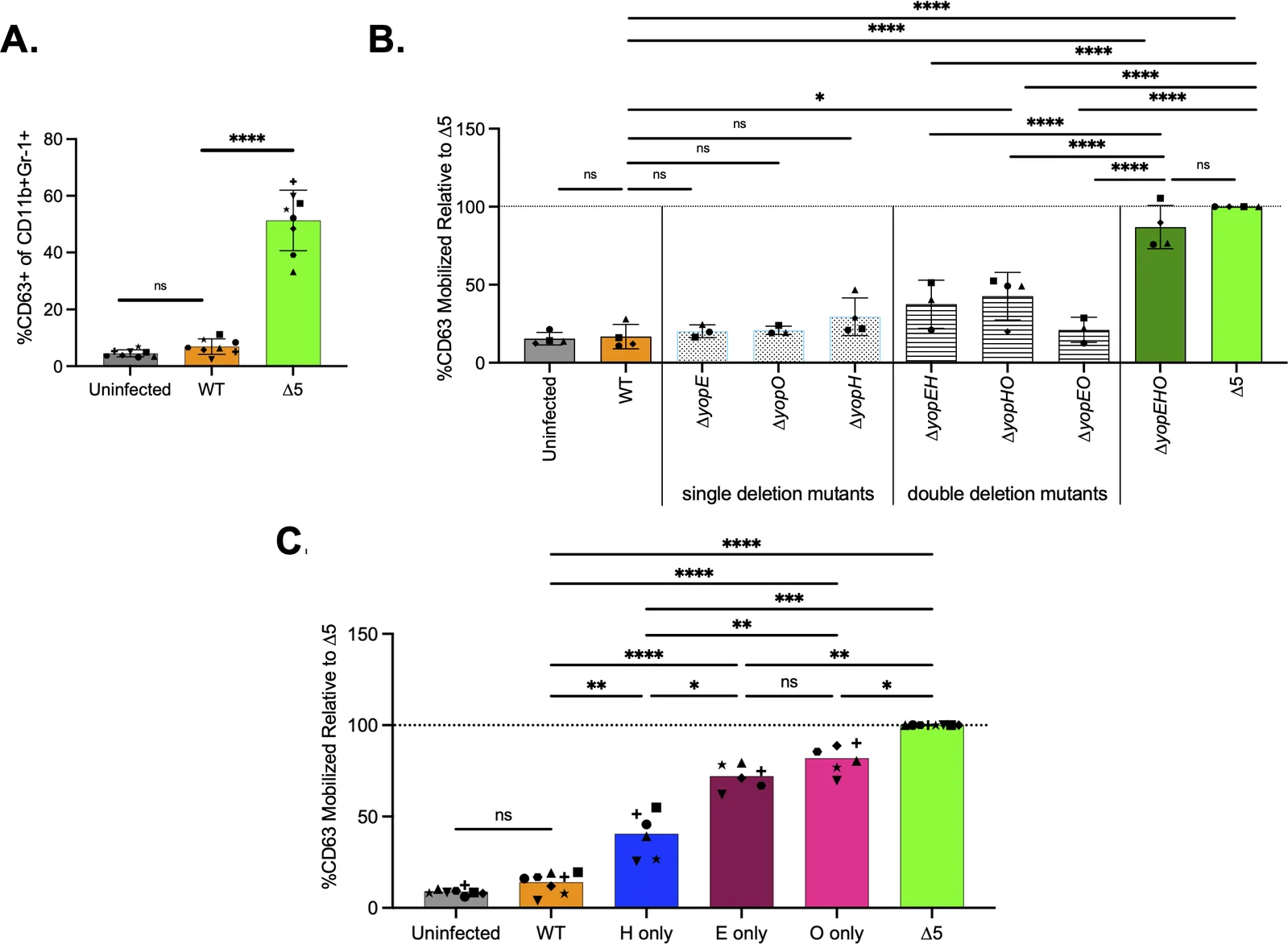
YopH, YopE, and YopO reduce CD63 plasma membrane localization after *Yptb* infection. (**A through C**) CG12-PMNs were infected with the indicated strains of *Yptb* at an MOI of 30 for 1 h at 37°C. ∆*5* is a strain of *Yptb* lacking the five Yop effectors: YopE, YopH, YopO, YopJ, and YopM. After infection, cells were stained, fixed, and analyzed on Aurora Cytek Spectral Cytometer. Gating strategy is shown in Fig. S1 (Fig. S1A and C). (**A**) Percentage of cells with CD63 localized to the plasma membrane is plotted. (**B and C**) Percentage of cells with CD63 localized to the plasma membrane plotted as a % of CD63 relative to ∆5, where ∆5 was set to 100%. Each dot is a biological replicate, and each biological replicate represents an average of 2–3 technical replicates. Bars are the mean ± SD of (**A and C**) 6–8 or (**B**) 3–5 biological replicates. Each dot represents a biological replicate, which are matched by symbol. Statistical significance was calculated using (**A and C**) one-way ANOVA or (**B**) mixed effects analysis followed by Tukey’s multiple comparison test. ns, not significant; *P*-value: *, <0.05; **, < 0.01; ***, <0.001; ****, <0.0001.

To measure the impact of specific Yops in inactivating distinct neutrophil signaling pathways, we monitored CD63 movement to the plasma membrane after infection with *Yptb* mutants. CD63 is a transmembrane protein found on several vesicles, including primary granules and extracellular vesicles (36, 37). Increased CD63 plasma membrane localization is widely used as a proxy to measure primary granule fusion with the plasma membrane (11, 12, 36). To establish which Yops were involved in blocking CD63 plasma membrane localization in our neutrophil cell system, we infected CG12-PMNs with WT-*Yptb* and various *yop* mutants (Fig. 2). The steady-state gMFI of CD63 in uninfected cells is higher than the CD63-KO due to trafficking of CD63-containing vesicles to and from the plasma membrane (Fig. S1C; Fig. 2A) (38). As expected, infection with a *Yptb-*∆*5* mutant, which lacks the genes encoding for five Yop effectors (YopE, YopM, YopO, YopJ, and YopH) or a *Yptb* ∆6 mutant (a ∆5 mutant with subsequent deletion of the gene encoding for YopK), triggered high levels of CD63 plasma membrane localization in greater than 50% of the cell population (Fig. 2A; Fig. S1C and Fig. S2). Infection of CG12-PMN with WT-*Yptb*, however, resulted in comparable levels of CD63 plasma membrane localization to uninfected cells, indicating that WT-*Yptb* blocks CD63 plasma membrane localization effectively (Fig. 2A; Fig. S1C). Thus, ER-HoxB8-derived PMNs behaved like PMNs isolated from humans and mice, where one or more Yops block CD63 movement to the plasma membrane (10–12).

To further interrogate the roles of specific Yops, we infected CG12-PMNs with a series of single, double, and triple *yop* deletion mutant strains. Infection with a triple deletion mutant, ∆*yopEHO,* induced levels of CD63 plasma membrane localization comparable to *Yptb-*∆*5-*infected levels (Fig. 2B). This demonstrated that YopE, YopO, and/or YopH play roles in reducing mobilization of CD63-expressing vesicles to the surface and indicated that the combined activities of YopM and YopJ play modest if any roles in preventing CD63 movement (Fig. 2B). Infection of CG12-PMNs with ∆*yopE*, ∆*yopO*, or ∆*yopH* single deletion mutants resulted in blockage of CD63 plasma membrane localization comparable to infection with WT-*Yptb* (Fig. 2B). This result pointed to functional redundancies among YopH, YopE, and YopO. None of the double deletion mutants tested (∆*yopEH*, ∆*yopHO*, or ∆*yopEO*) restored CD63 plasma membrane localization to *Yptb-*∆*5* or ∆*yopEHO* infected levels (Fig. 2B). These data demonstrated that any of the three Yops are sufficient to block CD63 plasma membrane localization and that no single Yop is necessary for this blockage.

To assess if YopH, YopE, or YopO were sufficient to block or reduce CD63 movement to the plasma membrane after *Yptb* infection, we infected CG12-PMNs with strains of *Yersinia* expressing a single Yop from their native locus in a *Yptb-*∆*5* background. These strains are referred to as “Yop-only” strains and were confirmed to secrete the indicated effector Yop (Fig. S3). Infection with “H-only” (∆*yopEMOJ*) resulted in the most potent blockage of CD63 plasma membrane localization to approximately half of the CD63 mobilization observed in a *Yptb-*∆5 infection (Fig. 2C). Infection with “E-only” (∆*yopMOJH*) or “O-only” (∆*yopEMJH*) resulted in modest but significant blockages of primary granule mobilization. Overall, no Yop was sufficient to completely block CD63 plasma membrane localization. Collectively, these data indicate that multiple overlapping pathways are involved in WT-*Yptb* blocking the trafficking of CD63-expressing vesicles to the plasma membrane, and these pathways involve those targeted by YopH, YopE, and YopO.

### Generation of knockout cell lines in Cas9-GFP-ER-HoxB8 myeloid progenitor cells

The observation that YopH, YopE, and YopO each partially reduced CD63 plasma membrane localization and that only a triple deletion strain of all three failed to suppress CD63 plasma membrane localization implies non-overlapping roles for each Yop. We hypothesized that inactivation of a Yop-targeted pathway and inactivation of another pathway (targeted by a different Yop) through deletion of the targeted gene in PMNs could delineate distinct pathways targeted by the Yops. We used the CG12 clonal cell line to create an array of KO cell lines targeting genes that encoded for proteins that are known to be or potentially targeted by YopE, YopH, and YopO. These include small Rho-GTPases (YopE and YopO targets), tyrosine kinases and adaptor proteins (YopH and YopO targets), and several genes critical for antimicrobial functions of PMNs. CRISPR inactivation of the targeted genes appeared highly efficient, as observed with the CD63-KO cell lines, where transduction of two distinct sgRNAs targeting *Cd63* resulted in comparable levels of surface CD63 to unstained controls (Fig. S4A).

As a preliminary screen, the newly generated array of KO pools was infected with *Yptb-*∆*5* and assessed for CD63 plasma membrane localization. Multiple KO cell lines showed reduced CD63 mobilization after *Yptb-*∆*5* infection, the majority of which encode for proteins downstream of integrin receptor activation (Fig. S4B). Importantly, in most cases where genes were targeted with two or more sgRNAs, the generated KO pools phenocopied one another. To minimize the likelihood of any WT cells in the KO pools from outcompeting the KOs during passaging, cells from 13 of these pools were single-cell cloned, validated for lack of protein expression, and used for further analyses. These 13 single-cell clones were chosen because the targeted genes encode proteins known to be (*Plcg2, Syk, Slp76, Rhog, Vav1, Skap2, Pxn*) or proteins potentially (*Btk, Lyn, Pyk2*) involved in YopH- or YopE-targeted pathways or because they encode critical for PMN antimicrobial functions (*Cybb, Mpo*). Eleven of the 13 targeted KO genes were confirmed using Western blot (Fig. S5), while the remaining two targeted KO genes, *Cybb-*KO-PMN and eGFP-KO, were confirmed using functional assays (Fig. S6). The *Cybb-*KO-PMN clones tested were unable to undergo release of extracellular ROS in response to PMA, which activates PKC downstream of receptor stimulation (Fig. S6A). Clones targeted for *eGFP* deletion showed lack of GFP expression via flow cytometry (Fig. S6B).

### Identifying key proteins involved in CD63 plasma membrane localization in response to *Yptb-*∆*5*

**Fig 3:**
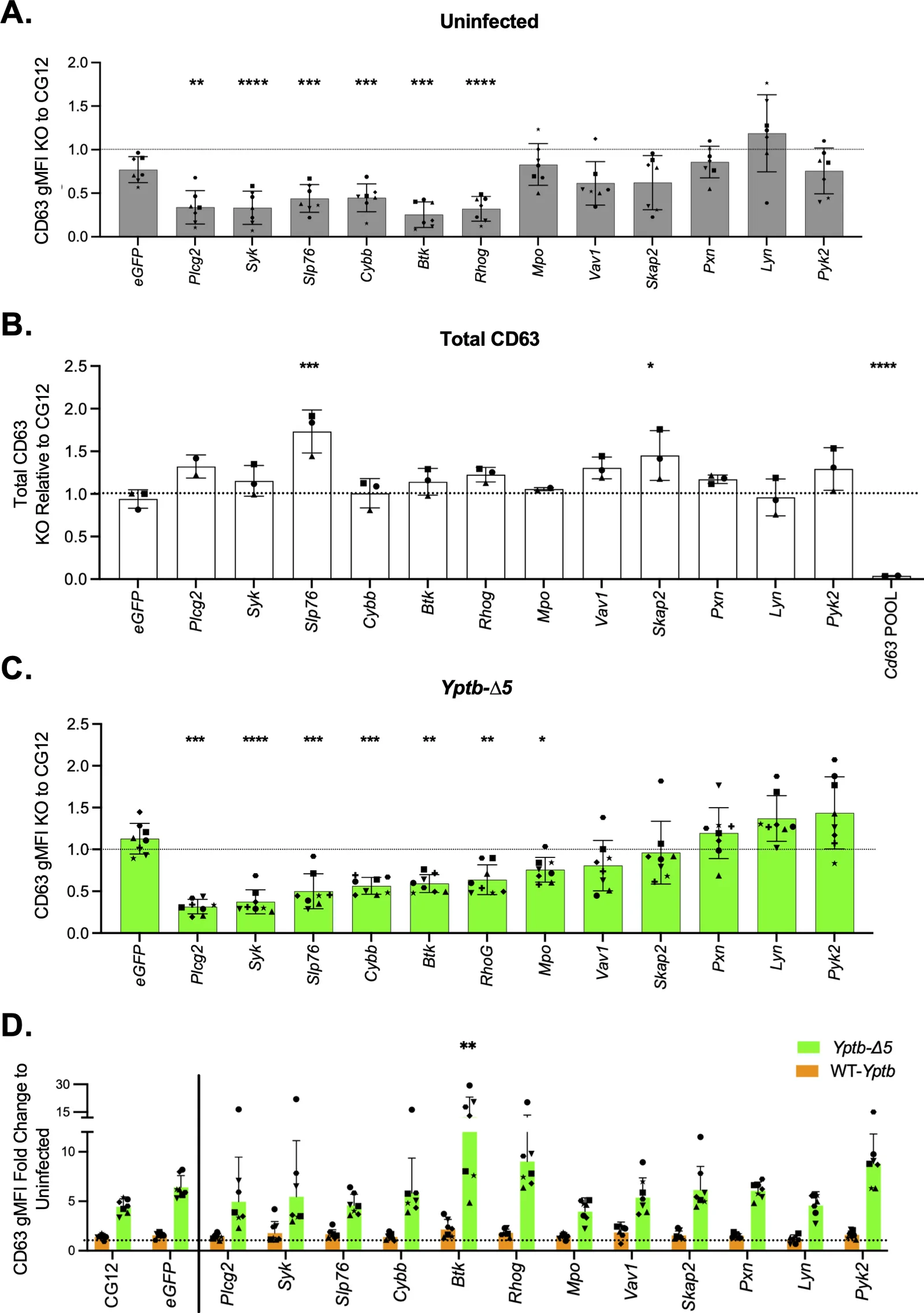
Identifying key proteins involved in CD63 plasma membrane localization in response to *Yptb-*∆*5.* (**A through D**) CG12-PMNs and single-cell cloned KO-PMNs were (**A, B, and D**) uninfected, (**C and D**) infected with *Yptb-*∆*5* (green), or (**D**) WT-*Yptb* (orange) with an MOI of 30 for 1 h at 37°C. (**A through D**) Cells were stained, fixed, and analyzed on Aurora Cytek Spectral Cytometer. Gating strategy is presented in Fig. S1 (Fig. S1A). CD63 geometric mean fluorescence intensity (gMFI) of each knock out cell line was normalized as follows: (**B**) cells were permeabilized prior to staining for total CD63 expression, (**A and B**) CD63 gMFI of uninfected KO cell line was divided by CD63 gMFI of uninfected CG12, (**C**) CD63 gMFI of the *Yptb-*∆*5*-infected KO cell line was divided by CD63 gMFI of the *Yptb-*∆*5-*infected CG12, (**D**) CD63 gMFI of infected conditions were normalized to uninfected CD63 gMFI of that same cell line to determine the fold change in CD63 surface expression. (**A, C, and D**) *n* = 7–8. (**B**) *n* = 2–3. Statistical analysis was performed by comparing eGFP-KO with other KO cell lines using one-way ANOVA followed by Dunnett’s test. Data are mean ± SD. Symbols are matched per biological replicate. *P* values: *, <0.05; **, <0.01; ***, <0.001; ****, <0.0001.

To assess how the absence of the CRISPR-targeted genes impacted basal levels of CD63 plasma membrane localization, we measured the amount of surface-localized CD63 in the uninfected panel of KO-PMN cell lines and compared it to the parental CG12-PMN cell line. An eGFP-KO single-cell clone served as a control cell line that has undergone gene editing but should not impact any neutrophil function. In the absence of infection, there were lower levels of CD63 plasma membrane localization in many of the uninfected cells, including PLCγ2-KO, SYK-KO, SLP-76-KO, CYBB-KO, BTK-KO, and RhoG-KO (Fig. 3A). To evaluate whether the variable levels of CD63 on the surface of the cell were a result of changes in total amounts of CD63 protein in the KO cell lines, the levels of CD63 in permeabilized KO-PMNs were measured by flow cytometry. Total levels of CD63 were all comparable to or slightly higher than those in eGFP-KO or CG12-PMN; however, these levels were not statistically significant except for SLP-76-KO and SKAP2-KO*,* which were higher than the parental cell line (Fig. 3B). This result eliminated the possibility that lower basal levels of CD63 at the membrane resulted from lower overall levels of CD63 and suggests that these genes are all involved in normal membrane trafficking of CD63-containing vesicles to the plasma membrane.

We next evaluated whether the absence of the targeted gene in the single-cell clones impacted CD63 movement to the plasma membrane after infection with *Yptb-*∆5 (Fig. 3C). In this case, we compared the amount of CD63 on the cell surface of a knockout strain to the amount on CG12-PMN after infection with *Yptb-*∆5. The absence of *Syk, Plcg2, Slp76,* and *Btk* demonstrated a significant defect in CD63 mobilization after infection with *Yptb-*∆*5* compared to CG12-PMN and eGFP-KO (Fig. 3C), which is consistent with the fact that these proteins transmit signals downstream of β1 integrin activation. In addition, *Cybb* and *Rhog* also played important roles in regulating levels of CD63 on the membrane (Fig. 3C). These results were largely consistent with our observations of the pooled KO cell lines where CD63 plasma membrane levels were low after infection with *Yptb-*∆5 (Fig. S4B). However, in the case of *Cybb,* the low levels observed in the single-cell clone did not phenocopy the CYBB-KO pools generated from two different gRNAs or HoxB8-derived *Cybb^−/−^* PMNs generated from *Cybb*^−/−^ mice (Fig. 3A and C; Fig. S4B and C). Overall, these data identified key proteins involved in CD63 plasma membrane localization in response to infection with *Yptb-*∆5, which includes RhoG*,* as well as key proteins that function downstream of integrins: PLCγ2, SYK, SLP76, and BTK (Fig. 3A and C; Fig. S4B and C).

Finally, we calculated the increase in CD63 at the plasma membrane after infection with *Yptb-*∆5 compared to its basal level in each cell line (Fig. 3D). Retention of some CD63 mobilization was critical for our next step in determining whether single Yops could better retard mobilization in specific KO cell lines. Infection of CG12-PMN and eGFP-KO with *Yptb-*∆*5* resulted in four- to sixfold increase in plasma membrane-localized CD63 compared to their uninfected levels. With the exception of BTK-KO, infection of all the KO single-cell clones with *Yptb-*∆*5* resulted in a 4- to 10-fold increase of CD63 compared to their baseline uninfected levels (Fig. 3D). BTK-KO had a 12-fold increase which was significantly higher although it is notable that the basal CD63 plasma membrane level was particularly low in BTK-KO (Fig. 3A). These data demonstrate that, compared to their respective baseline levels, all KO cell lines were able to mobilize CD63 to comparable or greater levels as the control cell lines in response to infection with *Yptb-*∆*5* (Fig. 3D). Moreover, the observation that knocking out these host proteins still resulted in mobilization of CD63+ vesicles after *Yptb-*∆*5* infection points to the use of multiple host pathways involved in primary granule mobilization.

### YopE and YopH target distinct signaling pathways to block CD63 plasma membrane localization

**Fig 4:**
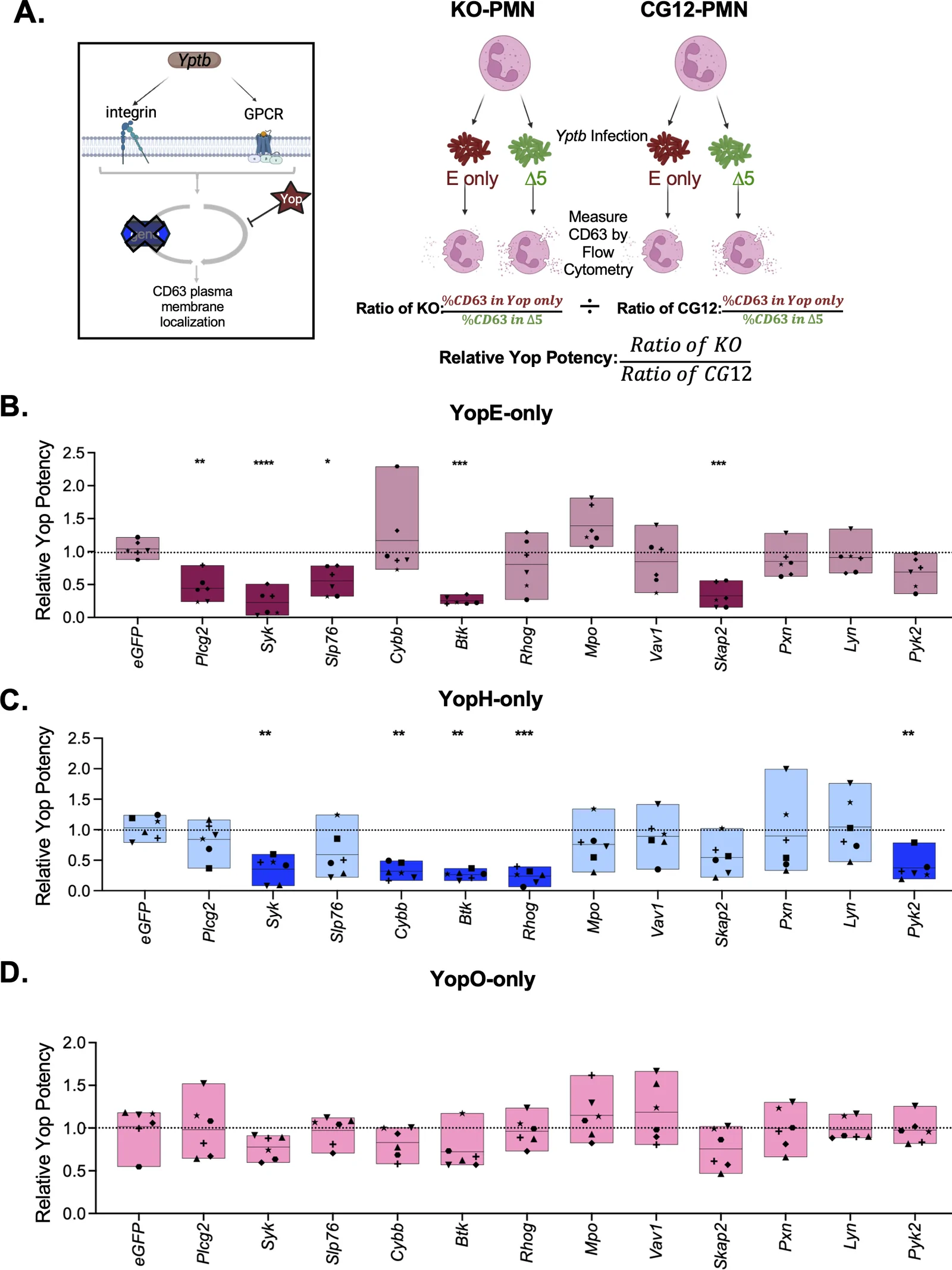
YopE and YopH target distinct signaling pathways to block CD63 plasma membrane localization. (**A**) Graphical representation of the model (left) and experimental design and setup for analysis (right). For each KO cell line, relative Yop potency was calculated by dividing %CD63+ of Yop-only-infected cells by %CD63+ of the ∆5-infected cell line. This ratio was divided by the same ratio obtained from the parental CG12-PMN. The normalized CD63 of KO to CG12 is plotted as relative Yop potency in panels **B–D**). CG12 and KO cell lines were differentiated into KO-PMNs and infected with (**B**) YopE-only, (**C**) YopH-only, and (**D**) YopO-only with an MOI of 30 for 1 h at 37°C. After infection, cells were stained, fixed, and analyzed on Aurora Cytek Spectral Cytometer. Cells were gated as shown in Fig. S1, and the percentage of CD63+ cells of each knock out cell line was normalized. *N* = 6. Biological replicates are indicated by a symbol, and each biological replicate had 2–6 technical replicates. Statistical analysis was performed by comparing eGFP-KO with KO cell lines using RM one-way ANOVA followed by Dunnett’s test. Symbols are matched per biological replicate. Bars represent the minimum to maximum, and the line indicates mean. *P* values: *, <0.05; **, <0.01; ***, <0.001; ****, <0.0001.

Based on the observation that individual Yop expression strains, “Yop-only” strains, partially blocked CD63 mobilization, we reasoned that *Yptb-*∆*5* may induce CD63 mobilization through several distinct pathways upon stimulation of a variety of receptors (Fig. 4A). We next sought to identify PMN KOs that synergize with Yop-only strains in blocking CD63 plasma membrane localization relative to the Yop-only-infected CG12-PMN. We hypothesized that we may observe enhanced Yop potency in blocking CD63 mobilization that could be due to inactivation of two distinct pathways: (i) a Yop-targeted pathway and (ii) a second pathway inactivated by deletion of a PMN gene (Fig. 4A). Thus, we infected our KO cell lines with Yop-only strains. The impact of individual Yops on each KO cell line was quantified by calculating a ratio of CD63 plasma membrane localization in the Yop-only condition divided by the *Yptb-*∆*5-*infected condition. To compare the impact of the Yop in each KO cell line relative to the parental CG12-PMN, we divided the calculated ratio for each KO cell line by the ratio obtained from CG12-PMN (Fig. 4A). We referred to this normalized ratio as Relative Yop Potency, where a value less than 1 indicates enhanced potency of the Yop in the KO cell line compared to the parental CG12-PMN cell line. This would signify that the Yop-targeted pathway and the protein encoded by the knocked-out gene belong to distinct pathways. On the other hand, if the Yop has the same impact in the absence of the targeted gene compared to the parental CG12-PMN line, then the Relative Yop Potency should be around 1, indicating that the deletion of the PMN gene did not disrupt additional pathways to the ones targeted by the Yop. Such a result could arise because either the KO has no impact on CD63 mobilization or the KO is in the Yop-targeted signal transduction pathway.

Infection of the eGFP-KO control with the Yop-only strains resulted in comparable levels of blockage to the CG12-PMN parental cell line (Fig. 4B through D). Notably, infection of our panel of KOs with YopE-only revealed that YopE-only was significantly better at uniquely dampening CD63 plasma membrane localization in the absence of SKAP2, SLP-76, and PLCγ2 (Fig. 4B). This indicates that, when these pathways are absent or disrupted, YopE’s ability to inactivate the signaling for CD63 mobilization becomes more apparent. We and others have previously shown that SKAP2, SLP-76, and PLCγ2 are dephosphorylated in the presence of YopH (4, 7, 17, 39). This result suggests that YopE targets and inactivates a pathway that is independent of the SKAP2/SLP-76/PLCγ2 YopH-targeted pathway and provides genetic evidence that YopH and YopE function on separate pathways to reduce CD63 plasma membrane localization.

YopH-only was significantly better at uniquely dampening CD63 plasma membrane localization in the absence of RhoG, CYBB, and PYK2 (Fig. 4C). The small Rho GTPase, RhoG, is a known target of YopE (21, 22); therefore, this observation that YopH-only’s impact was enhanced in the absence of RhoG indicates that YopH inactivates a signaling pathway that is independent of the YopE-mediated pathway (21, 22). This genetic approach allowed for distinction between the YopE-targeted RhoG pathway and the YopH-targeted SKAP2/SLP-76/PLCγ2 pathway.

In contrast with YopE-only and YopH-only strains, infection with YopO-only did not further reduce CD63 plasma membrane localization in any of the KO-PMN cell lines. Both YopE-only and YopH-only were more effective at blocking the residual signaling for CD63 mobilization in the SYK-KO and BTK-KO, indicating that the impact of YopE and YopH is each enhanced when the pathway(s) propagated by these proteins is impaired. This demonstrates that the residual signaling in their absence still requires small Rho GTPases and/or tyrosine kinases, which YopE and YopH can block, respectively.

### YopE mitigates primary granule release and extracellular ROS production in a SKAP2-independent manner

**Fig 5:**
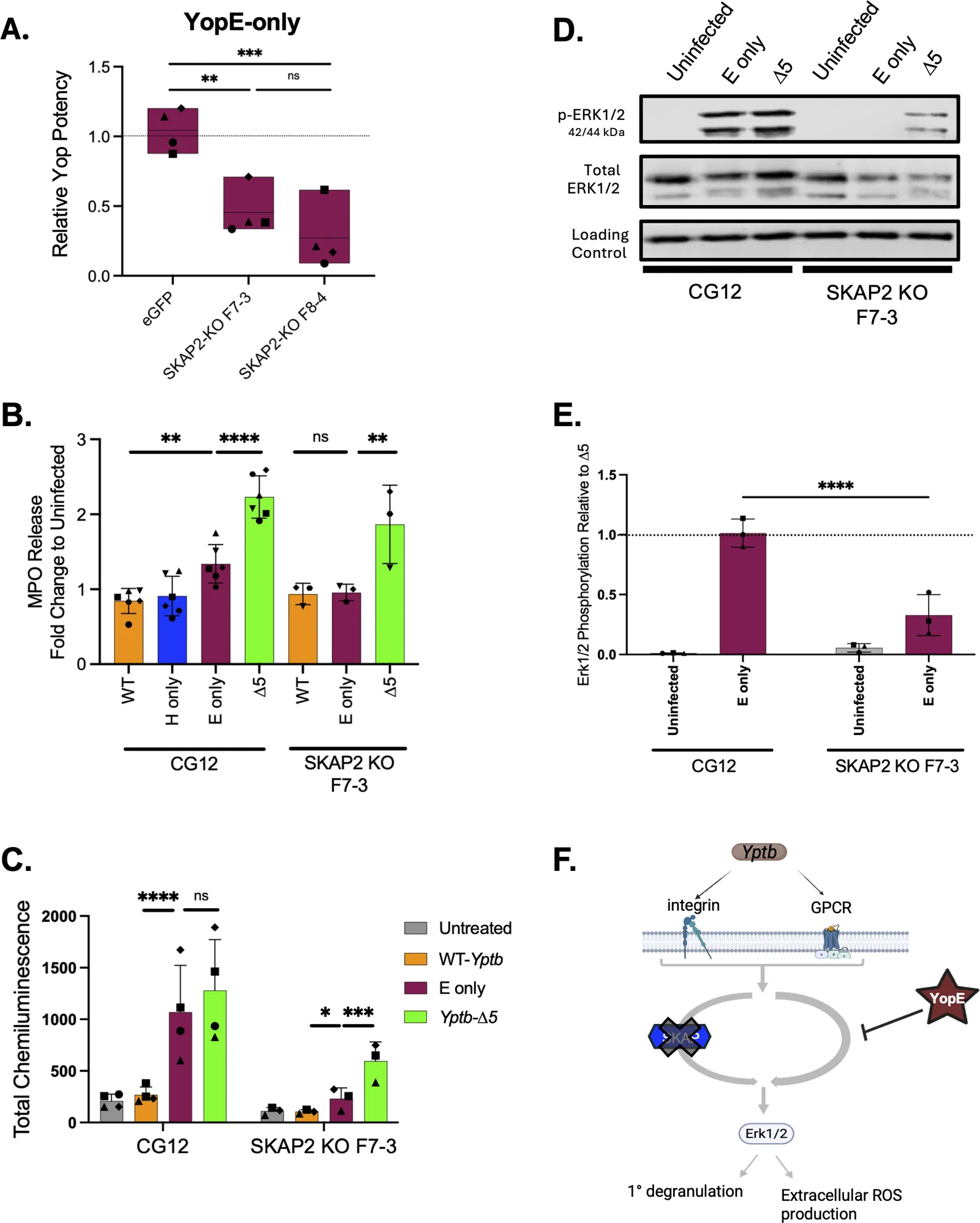
YopE mitigates primary granule release and extracellular ROS production in a SKAP2-independent manner. (**A through E**) CG12, eGFP-KO, SKAP2-KO F7-3, and (**A**) SKAP2-KO F8-4 were differentiated into PMNs. (**A**) Hoxb8-PMNs were infected with *Yptb* YopE-only and *Yptb-*∆*5* at an MOI of 30 for 1 h. Cells were stained, fixed, and analyzed on Aurora Cytek Spectral Cytometer. Cells were gated as shown in Fig. S1A and C. CD63+ of each knockout cell line was normalized as detailed in Fig. 4A. Statistical analysis was performed against eGFP using one-way ANOVA followed by Dunnett’s multiple comparisons test. (**B through E**) Cells were infected with the indicated *Yptb* strains at an MOI of 20 for (**B**) 1 h, (**C**) 30 min, and (**D and E**) 5 min. (**B**) After 1 h of infection, supernatant was collected to quantify MPO released via ELISA. The concentration of MPO in each sample was divided by the concentration detected in the uninfected condition of each respective cell line and reported as fold-change. The average of 3–6 biological replicates, with two technical replicates, is plotted. Statistical significance was determined using mixed-effects analysis followed by Dunnett’s test against E-only condition. (**C**) Extracellular ROS production was measured via an isoluminol chemiluminescence assay. Chemiluminescence was recorded every 2 min for 30 min of infection, and area under the curve (AUC) was calculated and normalized according to cell number using Cell Titer Glo. AUC values were log transformed, and statistical significance was calculated using two-way ANOVA followed by Tukey’s multiple comparison test. (**B through D**) Each symbol represents one biological replicate with the same symbol indicating samples were collected on the same day. (**D and E**) Lysates were immunoblotted with anti-phospho-Erk1/2; anti-RhoGDI was used as a loading control for all blots. Phospho-blots were stripped with 7M Guanidine HCl and probed with anti-Erk1/2 as a control. (**D**) Representative blot of quantified biological replicates in (**E**). (**E**) p-ERK1/2 signal for each biological replicate was normalized to RhoGDI for each experiment. The values from uninfected and E-only were then divided by the value from *Yptb-*∆*5* for each respective cell line. Normalized Erk1/2 phosphorylation relative to ∆5 is plotted. *n* = 3. Statistical significance was determined using two-way ANOVA followed by Sidak’s multiple comparison test. (**F**) Summary model. *P* values: *<0.05; **<0.01; *** <0.001; **** <0.0001.

Since CD63 mobilization was relatively unimpaired after infection with *Yptb*-Δ5 in SKAP2-KO PMNs (Fig. 3C), we sought to further understand the impact of YopE on antimicrobial responses in this host background. To further validate that YopE targets a SKAP2-independent pathway to inhibit CD63 plasma membrane localization, we assessed the impact of YopE on CD63 mobilization in an independent SKAP2-KO clone generated with a different sgRNA, F8-4. YopE-only had similar impacts on both SKAP2-KO single-cell clones (Fig. 5A). Since CD63 plasma membrane localization serves as a proxy for primary degranulation, we measured release of the primary granule component myeloperoxidase (MPO) in the supernatant of infected cells to directly measure primary granule release. WT-*Yptb* blocked MPO release to levels seen in uninfected CG12-PMN and SKAP2-KO, while *Yptb-*∆*5* infection resulted in a two-fold increase compared to uninfected (Fig. 5B). In CG12-PMN, infection with YopE-only reduced MPO release but was not sufficient to completely block MPO release to uninfected levels. However, in the absence of SKAP2*,* infection with YopE-only blocked MPO release to the same degree as the WT-*Yptb* condition (Fig. 5B). These results phenocopy the CD63 results and indicate that YopE-only inhibits primary granule mobilization and exocytosis in the absence of SKAP2. By contrast, infection with YopH-only effectively blocked MPO release but only reduced CD63 plasma membrane localization to about half of what was observed in the *Yptb-*∆*5*-infected CG12-PMN (Fig. 2C and Fig. 5B). This indicates that while YopH is sufficient to block release of primary granules completely, some CD63+ vesicles can still mobilize to the plasma membrane, demonstrating that CD63 mobilization does not always phenocopy levels of primary granule release. Furthermore, this shows that there are differences in the signaling events required for primary granule release and other processes resulting in CD63 plasma membrane localization, where YopH blocks release but not CD63 plasma membrane localization. By contrast, YopE blocks both processes in a SKAP2-independent manner.

Data from our lab have previously demonstrated that SKAP2 is required for maximal extracellular ROS production upon stimulation of integrin and GPCRs (4, 40). Here, we assessed whether YopE blocks extracellular ROS in the absence of SKAP2 after infection with WT-*Yptb*, YopE-only, or *Yptb-*∆5. SKAP2 was required for maximal extracellular ROS production after *Yptb-*∆*5* infection, but modest amounts of extracellular ROS were still detected in the absence of SKAP2 (Fig. 5C). YopE-only did not block ROS production in CG12-PMN as ROS levels were comparable to *Yptb*-∆5 (Fig. 5C). However, in the absence of SKAP2, infection with YopE-only significantly reduced the remaining extracellular ROS produced (Fig. 5C). Collectively, these data suggest that YopE mitigates primary granule release and extracellular ROS in a SKAP2-independent manner.

To evaluate the impact of YopE in the SKAP2-independent pathway relative to distal signaling events, we assessed the activation of extracellular regulated kinase 1/2 (ERK1/2). In WT-PMNs, SKAP2 is important for ERK1/2 activation, which leads to p47^phox^ translocation, activation of the NADPH oxidase complex, and ROS production (4, 41, 42). In the absence of SKAP2, modest amounts of ERK1/2 activation were observed after infection with *Yptb-*∆*5* (Fig. 5D and E) (4). In a SKAP2-KO, infection with a YopE-only strain blocked ERK activation, demonstrating that the inhibitory activity of YopE occurs upstream of ERK activation (Fig. 5D and E). These results are consistent with the ROS data, where the modest amount of extracellular ROS produced in a *Yptb-*∆*5*-infected SKAP2-KO was reduced by YopE. These data provide supporting biochemical evidence that YopE reduces ERK activation via a SKAP2-independent signal-transduction pathway that occurs upstream of ERK1/2 (Fig. 5F).

### ∆*yopE* is not restored for growth in *Skap2^−/^*^−^ mice

**Fig 6:**
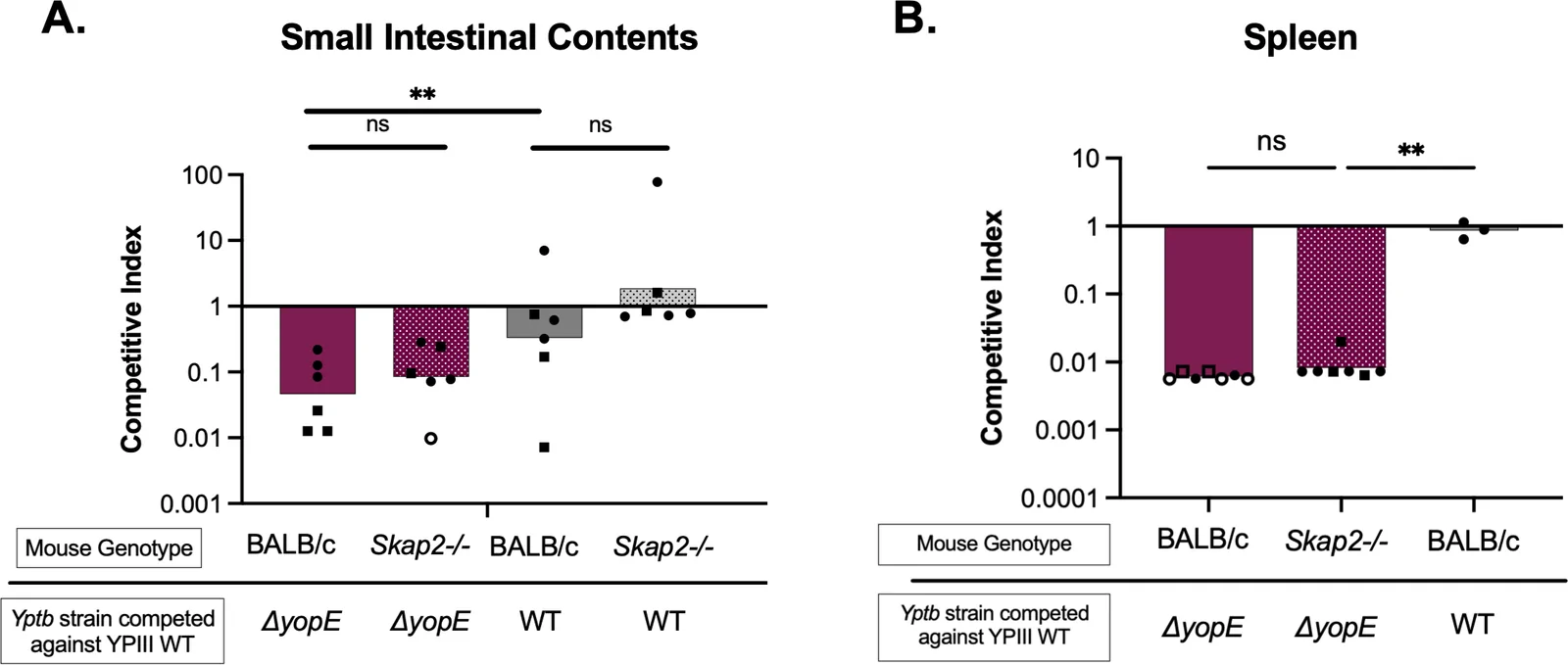
∆*yopE* is not restored for growth in *Skap2^−/−^* mice. (**A and B**) BALB/c WT and BALB/c *Skap2^−/−^* mice were (**A**) orally infected with equal doses of *Yptb* YPIII (kan^R^) and ∆*yopE* for a total CFU of 2 × 10^9^ or (**B**) intravenously infected with equal doses of *Yptb* YPIII (kan^R^) and ∆*yopE* for a total CFU of 1 × 10^3^. After 3 days of infection, (**A**) small intestinal contents or (**B**) spleen were harvested and homogenized, and outputs were plated on L plates containing irgasan. Outputs were then patched on L plates containing kanamycin to determine the competitive index (CI). Each dot represents a mouse. Open symbols indicate only WT^kan^ was detected in 100 colonies patched. CI values were log transformed, and statistical significance was calculated using one-way ANOVA followed by Dunnett’s comparison test. **, *P*-value < 0.01.

Since our findings demonstrate that in the absence of SKAP2, YopE inhibits PMN primary granule release, CD63 plasma membrane mobilization, and extracellular ROS production, we predicted that a ∆*yopE* mutant strain should not be restored for growth in a *Skap2*^−/−^ mouse. On the other hand, if YopE inactivates a pathway that requires SKAP2, we would expect that a ∆*yopE* mutant would be more competitive in a *Skap2*^−/−^ mouse and, thus, have a higher competitive index than it does in a BALB/c WT mouse. This result can be compared to previously published competition experiments with ∆*yopH*, which demonstrated that ∆*yopH* colonization is partially restored in the absence of *Skap2*, indicating that YopH targets a SKAP2-dependent process (4). To test this, BALB/c WT and *Skap2^−/^*− mice were orally (Fig. 6A) or intravenously (Fig. 6B) infected with equal doses of WT-*Yptb* (kan^R^) and ∆*yopE*. As expected, ∆*yopE* had a defect in colonizing BALB/c WT small intestinal contents upon oral infection (Fig. 6A) (20). While there was a slight trend toward ∆*yopE* being more competitive in the *Skap2*^−/−^ mouse, it was not significant, and the same trend held when the *Skap2*^−/−^ mouse was challenged with the isogenic WT-*Yptb* (kan^S^) vs the WT-*Yptb* (kan^R^). Intravenous administration of *Yptb* similarly demonstrated that ∆*yopE* was defective in colonizing the spleen in the systemic infection model in both the BALB/c WT and *Skap2*^−/−^ mice (Fig. 6B). Combined with our genetic and biochemical data, these tissue infection results provide an *in vivo* experimental model that supports our findings where YopE targets a SKAP2-independent pathway, ultimately mitigating the impact of PMNs on *Yptb*.

## DISCUSSION

**Fig 7:**
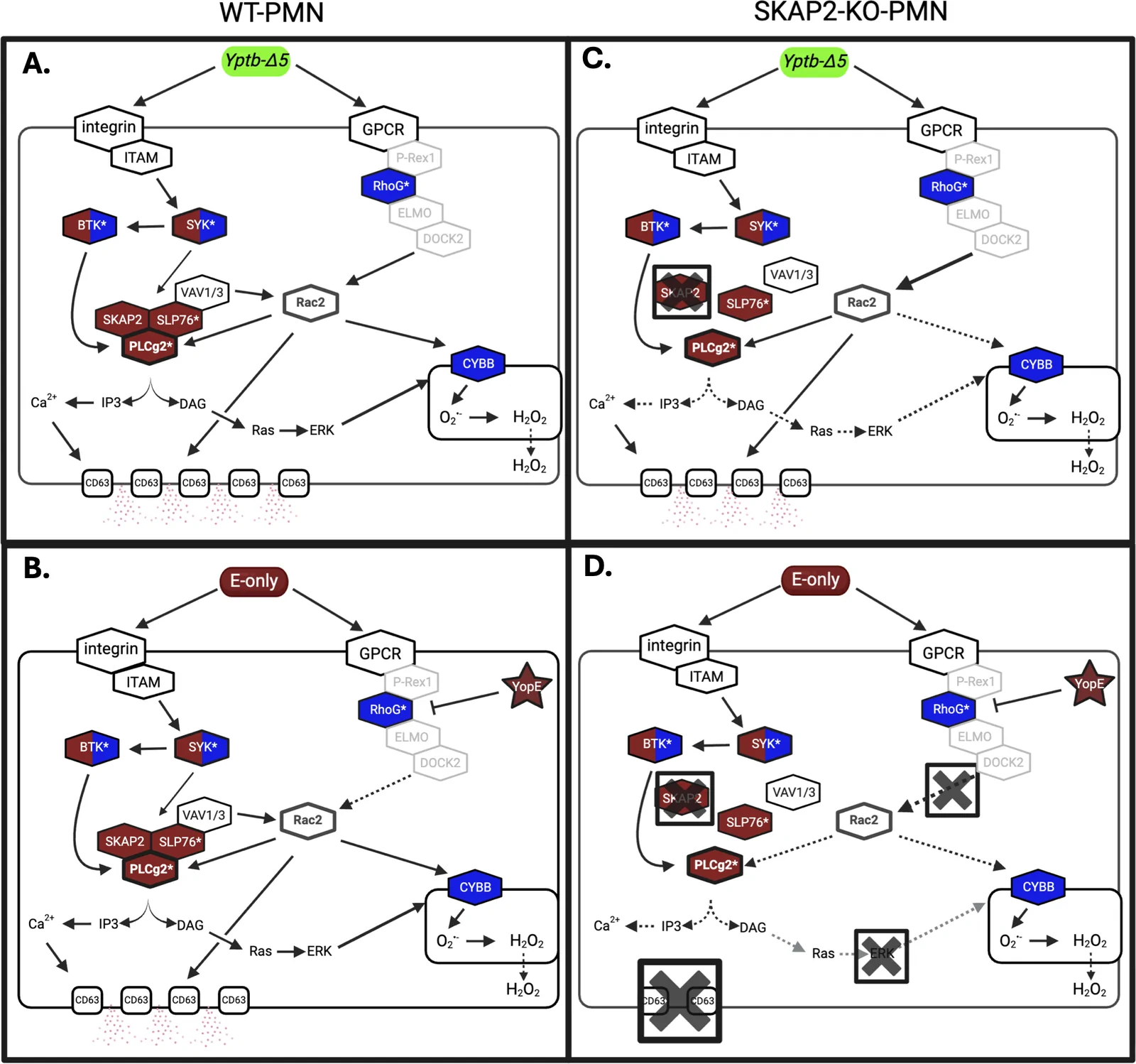
Proposed model. Our work identified proteins that play a critical role in CD63 plasma membrane localization upon *Yptb* infection (denoted with *). Two proteins in particular, Rac2 and PLCγ2 (thicker borders), play an important role in actin cytoskeletal rearrangements and Ca^2+^ flux, respectively, and are required for primary degranulation. During integrin outside-in signaling in WT-PMNs infected with *Yptb* ∆*5* (**A**)*,* ITAM bearing adaptors initiate downstream pathways involving recruitment and activation of SYK, leading to phosphorylation of BTK, as well as the formation of a signaling complex involving VAV1, SKAP2, SLP-76, and PLCγ2, which YopH can inactivate. PLCγ2 cleaves PIP2 into IP3 and DAG which can each induce Ca^2+^ flux and downstream activation of p47phox and subsequent activation of the NADPH oxidase complex via PKC-p38 and Ras-ERK pathways. The signaling complex containing VAV1 is involved in activating Rac2, which is critical for primary degranulation and ROS production. Another mechanism for Rac2 activation is likely through a GPCR-mediated pathway driven by RhoG. RhoG is activated by the GEF, P-Rex1, and upon activation can recruit ELMO/DOCK2, which functions as a GEF to activate Rac2. YopE (star symbol) can directly inactivate RhoG but only has a modest impact on blocking both primary degranulation and ROS in WT-PMNs (**B**). In the absence of the scaffolding protein SKAP2 (**C and D**), the protein complex with VAV1, SLP76, and PLCγ2 is impaired, and VAV1 can no longer activate Rac2. Thus, the activation of Rac2 becomes completely reliant on GPCR signaling via RhoG. In a SKAP2-KO, YopE can directly inactivate RhoG (**D**), subsequently impeding the activation of Rac2 and effectively inhibiting both primary degranulation and extracellular ROS production. In the absence of proteins colored in maroon, YopE-only was more potent in blocking CD63 plasma membrane localization. In the absence of proteins colored in blue, YopH-only was more potent in blocking CD63 plasma membrane localization. YopE and YopH were both more potent in the absence of proteins colored in both blue and maroon. Proteins outlined in gray were not tested in this work.

In this work, we developed and utilized a powerful system to generate genetic deletions in neutrophils and deconvolute the contributions of YopE and YopH in host neutrophil signaling pathways that converge on CD63 mobilization, a key marker of primary degranulation. This robust Cas9-ER-HoxB8 system can be used in screens to study neutrophil biology and applied to probe interactions of pathogens with neutrophils. Our findings indicate that multiple host pathways are triggered by *Yptb-*∆*5* to induce CD63 mobilization and primary granule mobilization, overall highlighting the crucial role of these host defenses to combat invading pathogens. Bacteria not equipped to inactivate these pathways, such as *K. pneumoniae*, are susceptible to neutrophil effector responses and are less able to withstand host immune responses, as demonstrated by CGD patients with increased susceptibility to this pathogen (43, 44). On the other hand, some pathogens, such as *Yersinia*, have evolved mechanisms to disarm different aspects of these signaling pathways. Thus, despite this multifaceted host response to *Yptb-*∆*5* infection, WT-*Yptb* effectively blocked CD63 plasma membrane localization in all KO cell lines (Fig. 3D)*,* demonstrating that *Yptb* is adeptly armed to halt CD63 mobilization. Additionally, this shows that the Yops in WT-*Yptb* effectively inhibit the residual pathways present in the KO cell lines. In HoxB8-PMNs, the absence of one or two of YopH, YopE, and YopO still resulted in significant blockage of CD63 mobilization and injection of YopH-only, YopE-only, or YopO-only partially blocked CD63 mobilization, indicating each Yop plays a role in preventing CD63 mobilization. On the host end, we identified RhoG and confirmed that PLCγ2, SYK, SLP76, and BTK all play central roles in the process of CD63 mobilization in response to *Yptb-*∆*5* infection (Fig. 7). Our findings also demonstrated that YopE’s contributions to blocking CD63 mobilization, primary granule release, and extracellular ROS production occur in an SKAP2-independent manner. Furthermore, YopE effectively prevented the residual signaling that remained in the absence of key proteins involved in integrin-mediated signaling (PLCγ2, SYK, SLP76, BTK) suggesting that YopE can block CD63 mobilization that is triggered by a non-integrin receptor.

YopE functions as a GAP for the small Rho GTPases RhoA, Rac1, Rac2, and RhoG (18–21, 45). In neutrophils, several small Rho GTPases tune actin cytoskeletal dynamics to permit primary granule mobilization (11, 23–26). The activation of Rac2 is required for CD63 plasma membrane localization (24). By contrast, RhoA inactivation is critical for granule translocation, which occurs by the host-encoded GAP, GMIP, and results in inhibition of F-actin assembly around the granules, thus facilitating granule translocation to the plasma membrane (26). Here, infection with YopE-only had a modest impact in blocking CD63 plasma membrane localization in the control cell lines CG12-PMN or eGFP-KO. Since Rac2 activation is essential for primary granule release (23, 25), YopE’s modest blockage of CD63 in CG12-PMN suggests that Rac2 is not a direct or the primary target in these parental cells. Notably, the inhibitory effect of YopE on CD63 mobilization and primary granule release was more pronounced in the absence of SKAP2. This indicates that in the absence of SKAP2, YopE either more efficiently targets Rac2, or more likely, that YopE is effectively targeting an upstream small Rho GTPase that leads to Rac2 activation.

We postulate that there are at least two distinct pathways driving the activation of Rac2 (Fig. 7). In WT-PMNs infected with *Yptb-*∆*5*, Rac2 is activated by VAV1/3 through a SKAP2-mediated mechanism, likely through its scaffolding function (Fig. 7). The absence of SKAP2 reveals the importance of a second pathway to activate Rac2, which we suspect is driven by RhoG via a GPCR-mediated pathway (Fig. 7). A role for RhoG is supported by our findings that the absence of RhoG resulted in a defect in steady-state levels of surface-localized CD63 as well as on CD63 mobilization upon infection with *Yptb-*∆*5* (Fig. 3). Damoulakis et al. have shown that there are RhoG-dependent and RhoG-independent mechanisms to activate Rac2 (46). Additionally, a previous study showed that the activation of Rac1 and Rac2 in neutrophils is severely impaired when the GEFs, VAV1 and P-Rex1, are both deleted compared to individual deletions of all members of the VAV family or P-Rex1, highlighting the two distinct pathways to activate Rac1/2 (47). P-Rex1 is a known GEF for RhoG and can activate Rac2 via ELMO/DOCK2 (46, 48). The fact that we observed no enhanced impact of YopE in the absence of VAV1 is likely because there is redundancy with VAV3, another member of the VAV family (49). In addition, while there were reduced levels of CD63 mobilization in the absence of BTK, PLCγ2, SYK, or SLP76, YopE was effective at blocking the residual signaling in these KO cell lines. Thus, we propose that when the signaling propagated by the protein microcluster formed by SKAP2-SLP-76-PLCγ2 is damaged genetically, the signaling for Rac2 activation becomes reliant on the RhoG-mediated pathway that may be triggered through the activation of one or more GPCRs that sense bacteria, and YopE impedes CD63 mobilization, primary degranulation, and ROS production by the inactivation of RhoG (Fig. 7).

YopH was the most potent single Yop at inhibiting CD63 mobilization and was completely effective at blocking MPO release. YopH inactivates host pathways governing Ca^2+^ influx and actin cytoskeletal rearrangements, both of which are important processes mediating primary degranulation (16). By inactivating PLCγ2, YopH inhibits cleavage of PIP_2_ into IP_3_ and DAG, effectively inhibiting Ca^2+^ influx and activation of extracellular ROS production, respectively (4, 14). YopH also targets the signaling complex involving VAV1 (14) for inactivation, thereby preventing the activation of Rac1/2 and inhibiting NADPH oxidase activation and actin cytoskeletal rearrangements required to traffic the granules to the plasma membrane. The potency of YopH is enhanced in the absence of RhoG, CYBB, and PYK2. One intriguing common phenotype of CYBB, RhoG, and PYK2 is that they all play roles in ROS generation (42, 50, 51). The possible link between generation of ROS and CD63 mobilization after *Yptb* infection remains to be explored experimentally. Studies in T cells have demonstrated that ROS can function as a signaling molecule to regulate cell signaling by transiently inactivating PTPases (52, 53). Thus, it is tantalizing to speculate that in the absence of host-generated ROS through genetic deletion of CYBB, RhoG, or PYK2, ROS-mediated inactivation of host and bacterial PTPase (YopH) is reduced. This could result in enhanced PTPase activity, and therefore, more potent inhibition of tyrosine kinase signal transduction relays occurs.

While YopH-only was sufficient to block MPO release, it did not block CD63 plasma membrane localization to the same levels as WT-*Yptb*. YopH’s enhanced potency in blocking CD63 mobilization in the absence of SYK, CYBB, BTK, RhoG, or PYK2 indicates that YopH targets pathways independent of these proteins, and these proteins are part of signal-transduction pathways that contribute to CD63 mobilization after *Yptb* infection. *Y. pestis* was recently shown to block release of neutrophil-derived EVs, which are enriched with CD63 (54, 55). In particular, YopH, along with YopE and YopK, played a role in altering proteins packaged in EVs (54). It is possible that YopH’s enhanced potency in reducing CD63 plasma membrane localization points to the involvement of these host proteins in regulating processes involved in CD63+ vesicle trafficking or uptake of EVs. EV biogenesis, uptake, and release of primary granules all involve vesicle trafficking and are at some level interconnected, but the mechanistic details and roles of these host proteins in these processes require further investigation.

Our work and others have demonstrated that SYK and BTK are critical for CD63 mobilization, yet residual CD63 mobilization occurs after infection with *Yptb-*∆*5* (56–58). SYK is a proximal protein downstream of integrin (58) and can recruit and phosphorylate BTK. Studies have shown that BTK activates the YopH target PLCγ2 (59, 60) and that BTK functions upstream of Rac2 (56). In this work, we found that both YopH-only and YopE-only infection led to enhanced blockage of CD63 mobilization in the absence of BTK and SYK. In the case of YopE, we suspect that SYK and BTK are not required to propagate the RhoG-mediated signaling, therefore, allowing for enhanced potency of YopE in this parallel or independent pathway (Fig. 7). On the other hand, in the SYK-KO and BTK-KO, the residual and impaired signaling mediated by the SKAP2-SLP-76-VAV1-PLCγ2 axis is effectively blocked by YopH, making YopH’s inhibitory effect more apparent.

Our observation that YopH, YopE, and YopO all contribute to blocking CD63 mobilization in ER-HoxB8 PMNs is largely consistent with previously published results in human and murine neutrophils, which showed that YopE and YopH were involved in blocking degranulation (4, 10–12). In addition to YopE and YopH, Pulsifer et al. also reported a role for YopJ and YopK, which we did not observe in our system (12). This could be because the *Y. pestis* KIM strain used by Pulsifer et al. has been shown to have a more active YopJ isoform in comparison to *Yptb* (61). Other possible explanations for the discrepancy could be a result of differences in the relative abundance of signal transduction proteins between human- and murine-derived PMNs or differences in receptors triggered between *Yptb* and *Y. pestis*.

In conclusion, the use of our dual-genetics approach demonstrates that there are at least two distinct host signaling responses which synergize to mobilize CD63 to the plasma membrane, one targeted by YopH and one by YopE. However, our panel of targeted genes did not provide additional insight into YopO’s targeted pathways, and a focus of our future studies is to uncover how YopO is contributing to this process. This work provides significant mechanistic insight into and untangles some of the complex receptor-mediated neutrophil signaling triggered upon infection with *Yptb*. Ultimately, we demonstrated how *Yersinia* YopE and YopH individually contribute to inactivate host signaling to block CD63 mobilization and ROS production. Finally, we developed a powerful system to generate genetic deletions in neutrophils, a key innate immune cell type that is notoriously difficult to manipulate genetically.

## MATERIALS AND METHODS

### Generation of Cas9-GFP-ER-HoxB8 cell line

ER-HoxB8 cells were generated according to previously described protocols with the following modifications (34, 40). To generate Cas9-GFP expressing ER-HoxB8 cells, the Spinflush method was used to harvest bone marrow cells from the femurs and tibias of 10-week-old C57BL/6J female mice (Rosa26-Cas9 knockin on B6J) from The Jackson Laboratory (Strain #26179). Briefly, one end of the bone was cut to expose the marrow and placed face down into a microcentrifuge tube. Bones in the microcentrifuge tube were spun at 10,000 × *g* for 30 s at room temperature (RT) to flush the marrow. Bone marrow cells were resuspended in flow cytometry buffer and passed through a 40-µm filter. Cells were then carefully layered over Lympholyte M (Cat# CL5031) and centrifuged at 1,000 × *g* for 20 min at RT. The cell suspension was then isolated, spun, and resuspended in RPMI + 10% (vol/vol) murine stem cell factor (mSCF) secreted from Chinese hamster ovary (CHO) cells + 10 ng/mL IL-3 + 10 ng/mL IL-6 and cultured for 24–48 h prior to transduction.

The ER-HoxB8 construct, MSCV-neo-HA-ER-Hoxb8, was packaged into a lentivirus (34). For transduction, bone marrow cells were seeded on a fibronectin-coated 12-well plate at 5 × 10^5^ per well and transduced via Spinoculation in the presence of 8 µg/mL polybrene at 1,000 × *g* for 90 min at RT. ER-HoxB8 cells were grown in GMP Media: complete RPMI (RPMI-1640, 10% FBS, 100 U/mL penicillin, and 0.1 mg/mL streptomycin) supplemented with 0.5 µM β-estradiol and 1.25% (vol/vol) mSCF. Successfully transduced cells with ER-HoxB8 were selected using 1 mg/mL G418. Cell lines derived from single-cell clones were generated by limiting dilution, and individual clones were screened for low CD11b baseline expression in undifferentiated cells. To ensure that Cas9 was functional in Cas9-GFP-ER-HoxB8 clones, cell lines were screened for Cas9 activity with a reporter plasmid (pXPR-011; [35]). Cas9-GFP single-cell lines were also assessed by flow cytometry for appropriate differentiation through surface expression of Gr-1 and CD11b or through cell cycle analysis with DyeCycle (Invitrogen Cat# V35003) after the removal of estrogen in the media. Single-cell clone, CG12, was selected for the parental cell line for the generation of KO cells.

### Differentiation of ER-HoxB8 cell lines into neutrophils

ER-HoxB8 cells were washed three times in PBS, prior to resuspending the cells at a concentration of 1.5 E5/mL. For 96-well plate differentiation, cells were resuspended in 200 µL of complete RPMI (cRPMI) + 1.25% (vol/vol) mSCF + 10 ng/mL IL-3 (PeproTech Cat# 213-13) +10 ng/mL granulocyte colony stimulating factor (GCSF) (PeproTech Cat# 300-23) and incubated for 48 h at 37°C in 5% CO_2_. After 48 h, cells were pelleted at 300 × *g* for 5 min and resuspended in 200 µL of cRPMI + 10 ng/mL GCSF for another 24-h incubation. For 6-well plate differentiation, 1.5 E5/mL of cells were resuspended in 6 mL of cRPMI + 1.25% (vol/vol) mSCF + 10 ng/mL IL-3 + 10 ng/mL GCSF and incubated for 48 h. After 48 h, cells were spun at 300 × *g* for 5 min and resuspended in 6 mL of cRPMI + 10 ng/mL GCSF.

### Generation of KO-ER-HoxB8 cell lines

To generate knockout (KO)-ER-HoxB8 cell lines (KO-HoxB8), CG12 was seeded into a 96-well plate at 50,000 cells/well. CG12 was then transduced with 30 µL of an array of custom sgRNA packaged in lentivirus (From Broad, see Table S1). Transduction was performed by spinfection of lentivirus at 1,000 × *g* for 60 min in the presence of 8 µg/mL polybrene (Sigma-Aldrich #107689). After spinfection, polybrene was diluted to a final concentration of 2 µg/mL. After 24 h, half of the medium was exchanged with GMP medium to further dilute the polybrene. To select for transduced Cas9-HoxB8 cells, cells were grown in GMP containing 2 µg/mL puromycin 36–48 h post-transduction, and puromycin was maintained in the medium for at least 72 h. Cells were then expanded, and selected cell lines were single-cell cloned by limiting dilution. KOs were confirmed using Western Blots, flow cytometry, or production of ROS.

### Infection of HoxB8-PMNs

After 72 h of growth in the absence of estrogen, PMNs were washed with PBS. For all washes, cells were pelleted at 300 × *g* for 5 min at RT. Cells were then incubated at RT for 30 min in HBSS^−/−^ (Gibco Cat# 14170-112). After 30 min, cells were pelleted at 300 × *g* for 5 min at RT, resuspended in HBSS^+/+^ (Gibco Cat# 14025-092), rested for 15 min at RT, and then shifted to 37°C for 15 min. For flow cytometry analysis, cells were seeded onto a 96-well round-bottom plate and infected with *Yptb* at an approximate MOI of 30:1 for 1 h at 37°C. After infection, cells were washed in PBS and processed for flow cytometry. For measurement of extracellular ROS, cells were seeded in a 96-well 4HBX plate (Co-star #3922) and infected with *Yptb* at an MOI of 20 for 30 min.

### Flow cytometry analysis

After infection, cells were washed in PBS at 4°C, prior to incubation with Fixable Viability Dye APC-eFluor780 (eBiosciences Cat# 65-0865-18) for 30 min on ice. For all washes, cells were pelleted at 300 × *g* for 5 min at 4°C. Cells were then washed with cold flow cytometry buffer (1% FBS in PBS) and blocked with mouse Fc block (BD Pharm Cat# 553142) for 10 min on ice according to the manufacturer’s protocol. Cells were then stained with antibodies for 30 min on ice: anti-CD11b-Pacific Blue (Biolegend Cat# 101224), anti-Gr-1 APC (Biolegend Cat# 108412), anti-CD63 PE (BD Pharm Cat# 564222), and CD62L Brilliant Violet 605 (Biolegend Cat# 104438). After staining, cells were washed once and fixed in 4% paraformaldehyde for 10 min at RT. After fixation, cells were washed in PBS and stored in flow cytometry buffer until analysis.

When screening cells in a 96-well format for flow cytometry (Fig. 3 and Fig. 4; Fig. S2), cells were analyzed using the BD Celesta (Fig. S2) or Cytek Aurora (Fig. 3 and Fig. 4) and analyzed with FlowJo Software (Version 10.10.0). For CD63 analysis, cells were gated as shown in Fig. S2. To compare the impact of specific Yops in our KO cell lines, we adapted an analysis performed in other published studies on gene interaction mapping and tailored it to our system (62, 63). For all KO cell lines tested, the percentage of cells with CD63 localized to the plasma membrane after infection with a Yop-only strain was divided by the plasma membrane localized CD63 after infection with the ∆5-*Yptb* strain, notated as CD63 $\frac{Yop_{ko}}{\Delta 5_{ko}}$. For each KO cell line, this ratio was then divided by the value obtained from the parental CG12 cell line after infection with the same strains on the same day (Fig. 4A). Relative Yop potency is plotted and calculated as


$$\frac{\left(\frac{Yop_{ko}}{\Delta 5{\;}_{ko}}\right)}{\left(\frac{Yop_{cg12}}{\Delta 5_{cg12}}\right)}$$

### Total levels of CD63 protein expression

Single-cell clones of each cell line were differentiated into Hoxb8-PMNs as described above. Cells were then permeabilized with 0.1% Triton X-100 prior to staining with PE-conjugated anti-CD63 and analyzed on Cytek Aurora Spectral Analyzer on the same day. Cells were gated as shown in Fig. S2, and CD63 gMFI was obtained for each KO cell line and divided by the gMFI of permeabilized CG12.

### Extracellular ROS using isoluminol

ROS isoluminol assays were performed as described previously (4, 40, 64) with the following modifications. Briefly, 4HBX white 96-well (Co-star #3922) plates were incubated and pre-coated with 10% FBS at 37°C for at least 1 h. Plates were washed three times with PBS prior to plating cells. Neutrophils were processed for infection as described above, and after incubating HoxB8-PMNs in HBSS^+/+^ for 15 min at 37°C, cells were loaded with 25 µM isoluminol (Sigma A8264) and 50 µg/mL HRP (Sigma P6782) and plated onto the pre-coated 4HBX 96-well plates. To synchronize infections, bacteria were spun onto the cells at 209 × *g* for 1 min at RT. Chemiluminescence was measured using a BioTek Synergy H1 microplate reader set to 37°C with 5% CO_2_ with readings taken every 2 min. To account for variations in cell density and viability between different cell lines, Promega Cell Titer Glo 2.0 (Cat# G9241) was used. When plating cells to measure ROS, cells were also plated in a separate well and mixed with a 1:1 ratio of Cell Titer Glo 2.0, where luminescence values of KO cell lines were divided by values from CG12. This ratio was then multiplied by the Total Chemiluminescence (AUC) from each condition.

### Western blot

For infected conditions, cells were seeded at 1.5 E6 cells per well on a 10% FBS-coated 24-well flat bottom plate (non-TC coated) and infected with an MOI of 20 for 5 min at 37°C 5% CO_2_. Infected cells (Fig. 5D) or clonal populations of cell lines assessed for the absence of protein (Fig. S4) were lysed with pre-heated 1× Novex lysis buffer (4× stock: 40% sucrose, 6.82% Tris-base, 6.66% Tris-HCl, 8% SDS, 0.06% EDTA, 0.075% Bromophenol Blue, 2.5 mM NaVO4, 100 mM DTT) and homogenized with pre-chilled Qiagen Qiashredders (Cat# 79656) at max speed in a microcentrifuge for 30 s at 4°C. Lysates were boiled for 10 min, and 2 E5 cells per lane were resolved on a 4%–12% Bis-Tris SDS-PAGE (Invitrogen #NP0322BOX). Infected samples were immunoblotted with anti-phospho-p44/42 MAPK (ERK1/2) (Thr202/Tyr204) (CST Cat# 9101s; 1:500 dilution) and probed with secondary antibody. To probe for total ERK, blots were stripped with 7 M guanidine HCl as previously described and probed with total anti-ERK1/2 (CST Cat# 9102s; 1:500) (40). Samples for KO validation were probed with ⍺-PLCγ2 (CST Cat# 3872S), ⍺-SYK antibody (Cat# 2712s), ⍺-SLP6 antibody (Cat# 05-1426), ⍺-Pyk2 (CST 3292s), ⍺-Lyn Ab (Cat# MA1-19334), ⍺-Vav1 monoclonal Ab (Upstate Cat 07-192), ⍺-MPO antibody (AbCam Cat# 9535), ⍺-BTK antibody (CST Cat# 8547S), ⍺-SKAP2 antibody (Proteinech Cat# 12926-1-AP), ⍺-RhoG antibody (SC-80015), or ⍺-PXN antibody (BD Pharm 610051). RhoGDI (CST Cat# 2564) or beta-actin was used as a loading control (SC-4778). Donkey anti-rabbit IRDye 800CW (LICOR Cat# 926-32213) or goat anti-mouse IRDye 800CW (LICOR Cat# 326-92910) secondary antibody was used at 1:20,000. Blots were imaged on Odyssey CLx Li-Cor system, and IS Image Studio was used for analysis and quantification.

### Measuring release of granules using ELISA

To assess the amount of granule proteins released, infections were performed as described for flow cytometry analyses, but supernatants were collected after 1 h of infection and stored at −80°C with Halt Protease and Phosphatase Inhibitors (Thermofisher Cat# 78442) until analysis. After thawing on ice, supernatants were diluted 1:30–1:50 in 1% BSA/PBS. myeloperoxidase (MPO) was measured using MPO DuoSet ELISA (R&D Systems; Cat# DY3667) according to the manufacturer’s protocol and quantified using a standard curve.

### Mouse infections

Female BALB/c 8- to 11-week-old mice (Charles River Laboratory) or *Skap2^−/−^* (4, 65) were orally infected with a 1:1 mixture of WT-*Yptb*^kan^ and *Yptb* ∆*yopE* via oral gavage with a total of 2E9 CFU in 100 µL PBS or intravenously infected with a total of 1,000 CFU in 100 µL PBS. Three days post-infection, mice were euthanized with CO_2_, tissues were harvested and homogenized, and outputs were plated on L plate with 0.5 µg/mL irgasan. Outputs were then patched on L^kan^ to determine competitive index by calculating a ratio, normalized to the following input:

$\frac{\left(\frac{\Delta {yopE}_{output}}{WTkan_{output}}\right)}{\left(\frac{\Delta {yopE}_{input}}{WTkan_{input}}\right)}$ .

### Bacterial strains and growth conditions

*Yptb* strains used are listed in Table 1. Strains constructed in this study were generated using allelic exchange as previously described (66, 67). Briefly, the Yop-only strains were constructed using previously published pCVD442 deletion constructs for *yopM* and *yopO* (67, 68). Primer pairs for the deletion constructs for *yopK* are listed in Table S2. Bacterial strains were cultured from a single colony plated on L irgasan plates that was grown overnight in 2×YT broth for 16–18 h at 26°C with aeration. For mouse infections, overnight cultures were used for infection, and kanamycin^r^ strains were grown in media supplemented with 50 µg/mL kanamycin. For infections of neutrophils, overnight cultures were diluted 1:40 in 2×YT supplemented with 20 mM MgCl_2_ and 20 mM sodium-oxalate. Cultures were incubated at 26°C for 1.5 h with aeration and shifted to 37°C for another 1.5 h with aeration. Bacteria were infected at the MOI indicated in specific experiments and in the figure legends.

**TABLE 1:** *Yersinia* strains used in this study

Original strain #	Description	Reference
JM301	YPIII pIB1	(69)
LL167	YPIII pIB1 ∆yopEMOJH (∆5)	(67)
ACF123	YPIII pIB1 ∆*yopEMOJ* (“H-only”)	This study
LL162	YPIII pIB1 ∆yopMOJH (“E-only”)	This study
MM48	YPIII pIB1 ∆yopEMJH (“O-only”)	This study
LL34	YPIII pIB1 ∆*yopO*	(67)
ACF125	YPIII pIB1 ∆*yopE*	(67)
ACF107	YPIII pIB1 ∆*yopH*	(67)
ACF108	YPIII pIB1 ∆*yopEH*	(67)
LL45	YPIII pIB1 ∆*yopEO*	(67)
LL57	YPIII pIB1 ∆*yopHO*	(67)
LL56	YPIII pIB1 ∆*yopEHO*	(67)
JM495	YPIII kanR	(70)
WS125c	YPIII ∆*yopE*	(20)
CMM048	YPIII ∆*yopK*	This work
CMM046	YPIII ∆*yopEMOJHK*	This work

### Statistical analyses

Statistical analysis was performed in GraphPad Prism Version 10, and statistical tests used are indicated in the figure legends.

## SUPPLEMENTAL MATERIAL

Fig. S1 mbio.01984-26-s0001.tiff Gating strategy.

Fig. S2 mbio.01984-26-s0002.tiff YopK is not necessary for blocking CD63 plasma membrane localization.

Fig. S3 mbio.01984-26-s0007.tiff Validating secretion of Yop proteins in Yop-only strains *Yptb* strains.

Fig. S4 mbio.01984-26-s0004.tiff Preliminary screen with pooled knockout cell lines.

Fig. S5 mbio.01984-26-s0005.tiff Validation of KO in single cell clones using Western blot.

Fig. S6 mbio.01984-26-s0006.tiff Validation of eGFP-KO and *Cybb^−/−^* clones.

Legends mbio.01984-26-s0003.tiff Supplemental figure legends.

Table S1 mbio.01984-26-s0008.docx sgRNA information.

Table S2 mbio.01984-26-s0009.pdf Primers used in study.

ASM does not own the copyrights to Supplemental Material that may be linked to, or accessed through, an article. The authors have granted ASM a non-exclusive, world-wide license to publish the Supplemental Material files. Please contact the corresponding author directly for reuse.

## References

[1] Isberg RR, Leong JM. 1990. Multiple beta 1 chain integrins are receptors for invasin, a protein that promotes bacterial penetration into mammalian cells. Cell 60:861–871. doi:10.1016/0092-8674(90)90099-z 2311122

[2] Sheahan KL, Isberg RR. 2015. Identification of mammalian proteins that collaborate with type III secretion system function: involvement of a chemokine receptor in supporting translocon activity. mBio 6:e02023-14. doi:10.1128/mBio.02023-14 PMC4337563 25691588

[3] Osei-Owusu P, Charlton TM, Kim HK, Missiakas D, Schneewind O. 2019. FPR1 is the plague receptor on host immune cells. Nature 574:57–62. doi:10.1038/s41586-019-1570-z PMC6776691 31534221

[4] Shaban L, Nguyen GT, Mecsas-Faxon BD, Swanson KD, Tan S, Mecsas J. 2020. *Yersinia pseudotuberculosis* YopH targets SKAP2-dependent and independent signaling pathways to block neutrophil antimicrobial mechanisms during infection. PLoS Pathog 16:e1008576. doi:10.1371/journal.ppat.1008576 PMC7241846 32392230

[5] Wren BW. 2003. The yersiniae--a model genus to study the rapid evolution of bacterial pathogens. Nat Rev Microbiol 1:55–64. doi:10.1038/nrmicro730 15040180

[6] Bliska JB, Wang X, Viboud GI, Brodsky IE. 2013. Modulation of innate immune responses by Yersinia type III secretion system translocators and effectors. Cell Microbiol 15:1622–1631. doi:10.1111/cmi.12164 PMC3788085 23834311

[7] Durand EA, Maldonado-Arocho FJ, Castillo C, Walsh RL, Mecsas J. 2010. The presence of professional phagocytes dictates the number of host cells targeted for Yop translocation during infection. Cell Microbiol 12:1064–1082. doi:10.1111/j.1462-5822.2010.01451.x PMC2906667 20148898

[8] Pechous RD, Sivaraman V, Price PA, Stasulli NM, Goldman WE. 2013. Early host cell targets of Yersinia pestis during primary pneumonic plague. PLoS Pathog 9:e1003679. doi:10.1371/journal.ppat.1003679 PMC3789773 24098126

[9] Marketon MM, DePaolo RW, DeBord KL, Jabri B, Schneewind O. 2005. Plague bacteria target immune cells during infection. Science 309:1739–1741. doi:10.1126/science.1114580 PMC3210820 16051750

[10] Taheri N, Fahlgren A, Fällman M. 2016. *Yersinia pseudotuberculosis* blocks neutrophil degranulation. Infect Immun 84:3369–3378. doi:10.1128/IAI.00760-16 PMC5116715 27620724

[11] Eichelberger KR, Jones GS, Goldman WE. 2019. Inhibition of neutrophil primary granule release during *Yersinia pestis* pulmonary infection. mBio 10:e02759-19. doi:10.1128/mBio.02759-19 PMC6904878 31822588

[12] Pulsifer AR, Vashishta A, Reeves SA, Wolfe JK, Palace SG, Proulx MK, Goguen J, Bodduluri SR, Haribabu B, Uriarte SM, Lawrenz MB. 2020. Redundant and cooperative roles for *Yersinia pestis* Yop effectors in the inhibition of human neutrophil exocytic responses revealed by gain-of-function approach. Infect Immun 88:e00909-19. doi:10.1128/IAI.00909-19 PMC7035916 31871100

[13] Montminy SW, Khan N, McGrath S, Walkowicz MJ, Sharp F, Conlon JE, Fukase K, Kusumoto S, Sweet C, Miyake K, Akira S, Cotter RJ, Goguen JD, Lien E. 2006. Virulence factors of Yersinia pestis are overcome by a strong lipopolysaccharide response. Nat Immunol 7:1066–1073. doi:10.1038/ni1386 16980981

[14] Rolán HG, Durand EA, Mecsas J. 2013. Identifying Yersinia YopH-targeted signal transduction pathways that impair neutrophil responses during *in vivo* murine infection. Cell Host Microbe 14:306–317. doi:10.1016/j.chom.2013.08.013 PMC3789382 24034616

[15] Andersson K, Carballeira N, Magnusson KE, Persson C, Stendahl O, Wolf-Watz H, Fällman M. 1996. YopH of *Yersinia pseudotuberculosis* interrupts early phosphotyrosine signalling associated with phagocytosis. Mol Microbiol 20:1057–1069. doi:10.1111/j.1365-2958.1996.tb02546.x 8809758

[16] Andersson K, Magnusson KE, Majeed M, Stendahl O, Fällman M. 1999. *Yersinia pseudotuberculosis*-induced calcium signaling in neutrophils is blocked by the virulence effector YopH. Infect Immun 67:2567–2574. doi:10.1128/IAI.67.5.2567-2574.1999 PMC116005 10225922

[17] Black DS, Marie-Cardine A, Schraven B, Bliska JB. 2000. The Yersinia tyrosine phosphatase YopH targets a novel adhesion-regulated signalling complex in macrophages. Cell Microbiol 2:401–414. doi:10.1046/j.1462-5822.2000.00061.x 11207596

[18] Black DS, Bliska JB. 2000. The RhoGAP activity of the *Yersinia pseudotuberculosis* cytotoxin YopE is required for antiphagocytic function and virulence. Mol Microbiol 37:515–527. doi:10.1046/j.1365-2958.2000.02021.x 10931345

[19] Von Pawel-Rammingen U, Telepnev MV, Schmidt G, Aktories K, Wolf-Watz H, Rosqvist R. 2000. GAP activity of the Yersinia YopE cytotoxin specifically targets the Rho pathway: a mechanism for disruption of actin microfilament structure. Mol Microbiol 36:737–748. doi:10.1046/j.1365-2958.2000.01898.x 10844661

[20] Songsungthong W, Higgins MC, Rolán HG, Murphy JL, Mecsas J. 2010. ROS-inhibitory activity of YopE is required for full virulence of Yersinia in mice. Cell Microbiol 12:988–1001. doi:10.1111/j.1462-5822.2010.01448.x PMC2897941 20148901

[21] Mohammadi S, Isberg RR. 2009. *Yersinia pseudotuberculosis* virulence determinants invasin, YopE, and YopT modulate RhoG activity and localization. Infect Immun 77:4771–4782. doi:10.1128/IAI.00850-09 PMC2772528 19720752

[22] Roppenser B, Röder A, Hentschke M, Ruckdeschel K, Aepfelbacher M. 2009. Yersinia enterocolitica differentially modulates RhoG activity in host cells. J Cell Sci 122:696–705. doi:10.1242/jcs.040345 19208761

[23] Abdel-Latif D, Steward M, Macdonald DL, Francis GA, Dinauer MC, Lacy P. 2004. Rac2 is critical for neutrophil primary granule exocytosis. Blood 104:832–839. doi:10.1182/blood-2003-07-2624 15073033

[24] Mitchell T, Lo A, Logan MR, Lacy P, Eitzen G. 2008. Primary granule exocytosis in human neutrophils is regulated by Rac-dependent actin remodeling. Am J Physiol Cell Physiol 295:C1354–65. doi:10.1152/ajpcell.00239.2008 PMC2878813 18799653

[25] Ilarraza R, Chao DV, Bodman JAR, Chesley A, Humble A, Shaheen F, Eitzen G, Lacy P. 2023. Rac2 regulates immune complex-mediated granule polarization and exocytosis in neutrophils. J Leukoc Biol 114:116–125. doi:10.1093/jleuko/qiad032 37017007

[26] Johnson JL, Monfregola J, Napolitano G, Kiosses WB, Catz SD. 2012. Vesicular trafficking through cortical actin during exocytosis is regulated by the Rab27a effector JFC1/Slp1 and the RhoA-GTPase-activating protein Gem-interacting protein. Mol Biol Cell 23:1902–1916. doi:10.1091/mbc.E11-12-1001 PMC3350554 22438581

[27] Aresta S, de Tand-Heim M-F, Béranger F, de Gunzburg J. 2002. A novel Rho GTPase-activating-protein interacts with Gem, a member of the Ras superfamily of GTPases. Biochem J 367:57–65. doi:10.1042/BJ20020829 PMC1222866 12093360

[28] Sugihara K, Nakatsuji N, Nakamura K, Nakao K, Hashimoto R, Otani H, Sakagami H, Kondo H, Nozawa S, Aiba A, Katsuki M. 1998. Rac1 is required for the formation of three germ layers during gastrulation. Oncogene 17:3427–3433. doi:10.1038/sj.onc.1202595 10030666

[29] Futosi K, Mócsai A. 2016. Tyrosine kinase signaling pathways in neutrophils. Immunol Rev 273:121–139. doi:10.1111/imr.12455 27558332

[30] Duquette PM, Lamarche-Vane N. 2014. Rho GTPases in embryonic development. Small GTPases 5:8. doi:10.4161/sgtp.29716 PMC4601498 25483305

[31] Liu Z, De Porto APNA, De Beer R, Roelofs JJTH, De Boer OJ, Florquin S, Van’t Veer C, Hendriks RW, Van der Poll T, De Vos AF. 2023. Bruton’s tyrosine kinase in neutrophils is crucial for host defense against *Klebsiella pneumoniae*. J Innate Immun 15:1–15. doi:10.1159/000524583 PMC10643901 35537415

[32] Moussavi-Harami SF, Cleary SJ, Magnen M, Seo Y, Conrad C, English BC, Qiu L, Wang KM, Abram CL, Lowell CA, Looney MR. 2024. Neutrophil-specific Shp1 loss results in lethal pulmonary hemorrhage in mouse models of acute lung injury. J Clin Invest 134:e183161. doi:10.1172/JCI183161 PMC11645157 39352872

[33] Shannon JG, Hinnebusch BJ. 2023. Characterization and CRISPR/Cas9-mediated genetic manipulation of neutrophils derived from Hoxb8-ER-immortalized myeloid progenitors. J Leukoc Biol 114:42–52. doi:10.1093/jleuko/qiad036 PMC10376455 36992528

[34] Wang GG, Calvo KR, Pasillas MP, Sykes DB, Häcker H, Kamps MP. 2006. Quantitative production of macrophages or neutrophils ex vivo using conditional Hoxb8. Nat Methods 3:287–293. doi:10.1038/nmeth865 16554834

[35] Doench JG, Hartenian E, Graham DB, Tothova Z, Hegde M, Smith I, Sullender M, Ebert BL, Xavier RJ, Root DE. 2014. Rational design of highly active sgRNAs for CRISPR-Cas9-mediated gene inactivation. Nat Biotechnol 32:1262–1267. doi:10.1038/nbt.3026 PMC4262738 25184501

[36] Kuijpers T, Tool A, van der Schoot C, Ginsel L, Onderwater J, Roos D, Verhoeven A. 1991. Membrane surface antigen expression on neutrophils: a reappraisal of the use of surface markers for neutrophil activation. Blood 78:1105–1111. doi:10.1182/blood.V78.4.1105.1105 1907873

[37] Andreu Z, Yáñez-Mó M. 2014. Tetraspanins in extracellular vesicle formation and function. Front Immunol 5:442. doi:10.3389/fimmu.2014.00442 PMC4165315 25278937

[38] Mathieu M, Névo N, Jouve M, Valenzuela JI, Maurin M, Verweij FJ, Palmulli R, Lankar D, Dingli F, Loew D, Rubinstein E, Boncompain G, Perez F, Théry C. 2021. Specificities of exosome versus small ectosome secretion revealed by live intracellular tracking of CD63 and CD9. Nat Commun 12:4389. doi:10.1038/s41467-021-24384-2 PMC8289845 34282141

[39] Black DS, Montagna LG, Zitsmann S, Bliska JB. 1998. Identification of an amino-terminal substrate-binding domain in the Yersinia tyrosine phosphatase that is required for efficient recognition of focal adhesion targets. Mol Microbiol 29:1263–1274. doi:10.1046/j.1365-2958.1998.01014.x 9767593

[40] Nguyen Giang T, Shaban L, Mack M, Swanson KD, Bunnell SC, Sykes DB, Mecsas J. 2020. SKAP2 is required for defense against *K. pneumoniae* infection and neutrophil respiratory burst. eLife 9:e56656. doi:10.7554/eLife.56656 PMC7250567 32352382

[41] Dewas C, Fay M, Gougerot-Pocidalo MA, El-Benna J. 2000. The mitogen-activated protein kinase extracellular signal-regulated kinase 1/2 pathway is involved in formyl-methionyl-leucyl-phenylalanine-induced p47phox phosphorylation in human neutrophils. J Immunol 165:5238–5244. doi:10.4049/jimmunol.165.9.5238 11046057

[42] Nguyen G.T., Green ER, Mecsas J. 2017. Neutrophils to the ROScue: mechanisms of NADPH oxidase activation and bacterial resistance. Front Cell Infect Microbiol 7:373. doi:10.3389/fcimb.2017.00373 PMC5574878 28890882

[43] Wolach B, Gavrieli R, de Boer M, van Leeuwen K, Berger-Achituv S, Stauber T, Ben Ari J, Rottem M, Schlesinger Y, Grisaru-Soen G, Abuzaitoun O, Marcus N, Zion Garty B, Broides A, Levy J, Stepansky P, Etzioni A, Somech R, Roos D. 2017. Chronic granulomatous disease: clinical, functional, molecular, and genetic studies. The Israeli experience with 84 patients. Am J Hematol 92:28–36. doi:10.1002/ajh.24573 27701760

[44] Bortoletto P, Lyman K, Camacho A, Fricchione M, Khanolkar A, Katz BZ. 2015. Chronic granulomatous disease: a large, single-center US experience. Pediatr Infect Dis J 34:1110–1114. doi:10.1097/INF.0000000000000840 PMC4568897 26181896

[45] Aili M, Isaksson EL, Hallberg B, Wolf-Watz H, Rosqvist R. 2006. Functional analysis of the YopE GTPase-activating protein (GAP) activity of *Yersinia pseudotuberculosis**.* Cell Microbiol 8:1020–1033. doi:10.1111/j.1462-5822.2005.00684.x 16681842

[46] Damoulakis G, Gambardella L, Rossman KL, Lawson CD, Anderson KE, Fukui Y, Welch HC, Der CJ, Stephens LR, Hawkins PT. 2014. P-Rex1 directly activates RhoG to regulate GPCR-driven Rac signalling and actin polarity in neutrophils. J Cell Sci 127:2589–2600. doi:10.1242/jcs.153049 24659802

[47] Lawson CD, Donald S, Anderson KE, Patton DT, Welch HCE. 2011. P-Rex1 and Vav1 cooperate in the regulation of formyl-methionyl-leucyl-phenylalanine-dependent neutrophil responses. J Immunol 186:1467–1476. doi:10.4049/jimmunol.1002738 21178006

[48] Katoh H, Negishi M. 2003. RhoG activates Rac1 by direct interaction with the Dock180-binding protein Elmo. Nature 424:461–464. doi:10.1038/nature01817 12879077

[49] Gakidis MAM, Cullere X, Olson T, Wilsbacher JL, Zhang B, Moores SL, Ley K, Swat W, Mayadas T, Brugge JS. 2004. Vav GEFs are required for beta2 integrin-dependent functions of neutrophils. J Cell Biol 166:273–282. doi:10.1083/jcb.200404166 PMC2172310 15249579

[50] Canino J, Guidetti GF, Galgano L, Vismara M, Minetti G, Torti M, Canobbio I. 2020. The proline-rich tyrosine kinase Pyk2 modulates integrin-mediated neutrophil adhesion and reactive oxygen species generation. Biochim Biophys Acta Mol Cell Res 1867:118799. doi:10.1016/j.bbamcr.2020.118799 32693110

[51] Condliffe AM, Webb LMC, Ferguson GJ, Davidson K, Turner M, Vigorito E, Manifava M, Chilvers ER, Stephens LR, Hawkins PT. 2006. RhoG regulates the neutrophil NADPH oxidase. J Immunol 176:5314–5320. doi:10.4049/jimmunol.176.9.5314 16621998

[52] O’Shea JJ, McVicar DW, Bailey TL, Burns C, Smyth MJ. 1992. Activation of human peripheral blood T lymphocytes by pharmacological induction of protein-tyrosine phosphorylation. Proc Natl Acad Sci USA 89:10306–10310. doi:10.1073/pnas.89.21.10306 PMC50327 1279675

[53] Kwon J, Qu C-K, Maeng J-S, Falahati R, Lee C, Williams MS. 2005. Receptor-stimulated oxidation of SHP-2 promotes T-cell adhesion through SLP-76-ADAP. EMBO J 24:2331–2341. doi:10.1038/sj.emboj.7600706 PMC1173147 15933714

[54] Sheneman KR, Cummins TD, Merchant ML, Hood JL, Uriarte SM, Lawrenz MB. 2025. Yersinia pestis actively inhibits the production of extracellular vesicles by human neutrophils. J Extracell Vesicles 14:e70074. doi:10.1002/jev2.70074 PMC12003101 40240908

[55] Escola JM, Kleijmeer MJ, Stoorvogel W, Griffith JM, Yoshie O, Geuze HJ. 1998. Selective enrichment of tetraspan proteins on the internal vesicles of multivesicular endosomes and on exosomes secreted by human B-lymphocytes. J Biol Chem 273:20121–20127. doi:10.1074/jbc.273.32.20121 9685355

[56] Desai JV, Zarakas MA, Wishart AL, Roschewski M, Aufiero MA, Donkò A, Wigerblad G, Shlezinger N, Plate M, James MR, et al.. 2024. BTK drives neutrophil activation for sterilizing antifungal immunity. J Clin Invest 134:e176142. doi:10.1172/JCI176142 PMC11178547 38696257

[57] de Porto AP, Liu Z, de Beer R, Florquin S, Roelofs JJTH, de Boer OJ, den Haan JMM, Hendriks RW, van ’t Veer C, van der Poll T, de Vos AF. 2021. Bruton’s tyrosine kinase-mediated signaling in myeloid cells is required for protective innate immunity during pneumococcal pneumonia. Front Immunol 12:723967. doi:10.3389/fimmu.2021.723967 PMC8450579 34552589

[58] Mócsai A, Zhou M, Meng F, Tybulewicz VL, Lowell CA. 2002. Syk is required for integrin signaling in neutrophils. Immunity 16:547–558. doi:10.1016/s1074-7613(02)00303-5 11970878

[59] Mueller H, Stadtmann A, Van Aken H, Hirsch E, Wang D, Ley K, Zarbock A. 2010. Tyrosine kinase Btk regulates E-selectin-mediated integrin activation and neutrophil recruitment by controlling phospholipase C (PLC) gamma2 and PI3Kgamma pathways. Blood 115:3118–3127. doi:10.1182/blood-2009-11-254185 PMC2858472 20167705

[60] Volmering S, Block H, Boras M, Lowell CA, Zarbock A. 2016. The neutrophil Btk signalosome regulates integrin activation during sterile inflammation. Immunity 44:73–87. doi:10.1016/j.immuni.2015.11.011 PMC5030078 26777396

[61] Zheng Y, Lilo S, Brodsky IE, Zhang Y, Medzhitov R, Marcu KB, Bliska JB. 2011. A Yersinia effector with enhanced inhibitory activity on the NF-κB pathway activates the NLRP3/ASC/caspase-1 inflammasome in macrophages. PLoS Pathog 7:e1002026. doi:10.1371/journal.ppat.1002026 PMC3080847 21533069

[62] Joshi SM, Pandey AK, Capite N, Fortune SM, Rubin EJ, Sassetti CM. 2006. Characterization of mycobacterial virulence genes through genetic interaction mapping. Proc Natl Acad Sci USA 103:11760–11765. doi:10.1073/pnas.0603179103 PMC1544243 16868085

[63] Halder V, McDonnell B, Uthayakumar D, Usher J, Shapiro RS. 2021. Genetic interaction analysis in microbial pathogens: unravelling networks of pathogenesis, antimicrobial susceptibility and host interactions. FEMS Microbiol Rev 45. doi:10.1093/femsre/fuaa055 33145589

[64] Dahlgren C, Karlsson A, Bylund J. 2007. Measurement of respiratory burst products generated by professional phagocytes. Methods Mol Biol 412:349–363. doi:10.1007/978-1-59745-467-4_23 18453123

[65] Alenghat FJ, Baca QJ, Rubin NT, Pao LI, Matozaki T, Lowell CA, Golan DE, Neel BG, Swanson KD. 2012. Macrophages require Skap2 and Sirpα for integrin-stimulated cytoskeletal rearrangement. J Cell Sci 125:5535–5545. doi:10.1242/jcs.111260 PMC3561861 22976304

[66] Mecsas J, Raupach B, Falkow S. 1998. The *Yersinia* Yops inhibit invasion of *Listeria*, *Shigella* and *Edwardsiella* but not salmonella into epithelial cells. Mol Microbiol 28:1269–1281. doi:10.1046/j.1365-2958.1998.00891.x 9680215

[67] Logsdon LK, Mecsas J. 2003. Requirement of the *Yersinia pseudotuberculosis* effectors YopH and YopE in colonization and persistence in intestinal and lymph tissues. Infect Immun 71:4595–4607. doi:10.1128/IAI.71.8.4595-4607.2003 PMC166012 12874339

[68] McCoy MW, Marré ML, Lesser CF, Mecsas J. 2010. The C-terminal tail of *Yersinia pseudotuberculosis* YopM is critical for interacting with RSK1 and for virulence. Infect Immun 78:2584–2598. doi:10.1128/IAI.00141-10 PMC2876544 20368345

[69] Portnoy DA, Wolf-Watz H, Bolin I, Beeder AB, Falkow S. 1984. Characterization of common virulence plasmids in *Yersinia* species and their role in the expression of outer membrane proteins. Infect Immun 43:108–114. doi:10.1128/iai.43.1.108-114.1984 PMC263395 6317562

[70] Mecsas J, Bilis I, Falkow S. 2001. Identification of attenuated *Yersinia pseudotuberculosis* strains and characterization of an orogastric infection in BALB/c mice on day 5 postinfection by signature-tagged mutagenesis. Infect Immun 69:2779–2787. doi:10.1128/IAI.67.5.2779-2787.2001 PMC98225 11292689

