# Supplementary material for: Neutrophil degranulation and extracellular ROS production are inactivated by Yersinia pseudotuberculosis YopE through a SKAP2 independent pathway
Source: mBio. 2026 Oct 6;17(11):e01984-26. doi: 10.1128/mbio.01984-26 (PMC13753789; doi:10.1128/mbio.01984-26)
Supplement: Supplementary file 1 [file mbio.01984-26-s0009.pdf]

Well	Clone ID	Target Sequence	Barcode Sequence	Vector	Search Taxon	On-target Gene ID:	On-target Gene Symbol	Orig. Target Taxo	Orig. Target Gene I	Orig. Target Gene Symb	Titer data
A01	BRDN0007709505	CACACTGAAGACACCCCACG	CACACTGAAGACACCCCACG	pXPR_050	10090	11689	Alox5	10090	11689	Alox5	10579412-maxOut
A02	BRDN0007709324	TTCTACAATGACTTCGAACG	TTCTACAATGACTTCGAACG	pXPR_050	10090	11689	Alox5	10090	11689	Alox5	10579412-maxOut
A03	BRDN0007709518	TGTGCGGCTAGTCATCCGGA	TGTGCGGCTAGTCATCCGGA	pXPR_050	10090	109689	Arrb1	10090	109689	Arrb1	10579412-maxOut
A04	BRDN0007709457	GTCTGCATACTGGCGCACTG	GTCTGCATACTGGCGCACTG	pXPR_050	10090	109689	Arrb1	10090	109689	Arrb1	10579412-maxOut
A05	BRDN0007709550	AATCCGGTACAATAGTGACC	AATCCGGTACAATAGTGACC	pXPR_050	10090	12229	Btk	10090	12229	Btk	10579412-maxOut
A06	BRDN0007709362	ATGAATATGACTTTGAACGT	ATGAATATGACTTTGAACGT	pXPR_050	10090	12229	Btk	10090	12229	Btk	10579412-maxOut
A07	BRDN0007709347	GATGTACAAGGACCGTATCG	GATGTACAAGGACCGTATCG	pXPR_050	10090	332579	Card9	10090	332579	Card9	255174.918
A08	BRDN0007709346	TCCATACATTCAAGTGCTGG	TCCATACATTCAAGTGCTGG	pXPR_050	10090	332579	Card9	10090	332579	Card9	5839721.737
A09	BRDN0007709464	GACAGGAAGATGGCAAACTG	GACAGGAAGATGGCAAACTG	pXPR_050	10090	12512	Cd63	10090	12512	Cd63	10579412-maxOut
A10	BRDN0007709341	CCTTCCTAGAATAACTGCTG	CCTTCCTAGAATAACTGCTG	pXPR_050	10090	12512	Cd63	10090	12512	Cd63	220866.3073
A11	BRDN0007709511	TCTGCGGATTGTAATCAGGA	TCTGCGGATTGTAATCAGGA	pXPR_050	10090	13035	Ctsg	10090	13035	Ctsg	8247589.538
A12	BRDN0007709451	GGGAACAAATGTACTCCAGG	GGGAACAAATGTACTCCAGG	pXPR_050	10090	13035	Ctsg	10090	13035	Ctsg	10579412-maxOut
B01	BRDN0007709528	TTTACCAGACGAATTGTACG	TTTACCAGACGAATTGTACG	pXPR_050	10090	13058	Cybb	10090	13058	Cybb	10579412-maxOut
B02	BRDN0007709342	CCTCTACCAAAACCATTCGG	CCTCTACCAAAACCATTCGG	pXPR_050	10090	13058	Cybb	10090	13058	Cybb	10579412-maxOut
B03	BRDN0007709503	GGCTTGGCAAACCATCCACG	GGCTTGGCAAACCATCCACG	pXPR_050	10090	242705	E2f2	10090	242705	E2f2	10579412-maxOut
B04	BRDN0007709439	AAGATTGGAAGTGCCGGACA	AAGATTGGAAGTGCCGGACA	pXPR_050	10090	242705	E2f2	10090	242705	E2f2	10579412-maxOut
B05	BRDN0007709530	TGTCGTTCAGCAGTTGTGAT	TGTCGTTCAGCAGTTGTGAT	pXPR_050	10090	50701	Elane	10090	50701	Elane	10579412-maxOut
B06	BRDN0007709404	CCGGCCACCAACAATCTCTG	CCGGCCACCAACAATCTCTG	pXPR_050	10090	50701	Elane	10090	50701	Elane	10579412-maxOut
B07	BRDN0007709506	TTGCTGCTCGGAGTTAACAG	TTGCTGCTCGGAGTTAACAG	pXPR_050	10090	23871	Ets1	10090	23871	Ets1	97653.64441
B08	BRDN0007709504	GAAGATCCTCGAATGACCTG	GAAGATCCTCGAATGACCTG	pXPR_050	10090	23871	Ets1	10090	23871	Ets1	10579412-maxOut
B09	BRDN0007709482	GGAGTCAAACTGGGCCCGTG	GGAGTCAAACTGGGCCCGTG	pXPR_050	10090	14191	Fgr	10090	14191	Fgr	10579412-maxOut
B10	BRDN0007709422	GGCGACGAATATGGTCACTC	GGCGACGAATATGGTCACTC	pXPR_050	10090	14191	Fgr	10090	14191	Fgr	282920.1423
B11	BRDN0007709445	CAAACAGCAACAATTGACAT	CAAACAGCAACAATTGACAT	pXPR_050	10090	14289	Fpr2	10090	14289	Fpr2	10579412-maxOut
B12	BRDN0007709325	GATGCAGAACACAAATGCAG	GATGCAGAACACAAATGCAG	pXPR_050	10090	14289	Fpr2	10090	14289	Fpr2	10579412-maxOut
C01	BRDN0007709555	AAGGTGTGTCTGGGAAATCG	AAGGTGTGTCTGGGAAATCG	pXPR_050	10090	23880	Fyb	10090	23880	Fyb	10579412-maxOut
C02	BRDN0007709368	GGCAGCGAAACCTACCTACG	GGCAGCGAAACCTACCTACG	pXPR_050	10090	23880	Fyb	10090	23880	Fyb	0-lowEnd
C03	BRDN0007709549	GTGGATACTATATCACAACG	GTGGATACTATATCACAACG	pXPR_050	10090	14360	Fyn	10090	14360	Fyn	10579412-maxOut
C04	BRDN0007709548	AGCCCGCTCCTTGACAACCG	AGCCCGCTCCTTGACAACCG	pXPR_050	10090	14360	Fyn	10090	14360	Fyn	10579412-maxOut
C05	BRDN0007709547	ACAGCAACAGCATGCCCCCA	ACAGCAACAGCATGCCCCCA	pXPR_050	10090	15162	Hck	10090	15162	Hck	10579412-maxOut
C06	BRDN0007709546	TCCATCTGGAGGGATTCTCG	TCCATCTGGAGGGATTCTCG	pXPR_050	10090	15162	Hck	10090	15162	Hck	10579412-maxOut
C07	BRDN0007709510	GAGTATAGGCAAATCCCGTG	GAGTATAGGCAAATCCCGTG	pXPR_050	10090	16414	Itgb2	10090	16414	Itgb2	10579412-maxOut
C08	BRDN0007709509	ATCCTGAGTTCGACCAACGG	ATCCTGAGTTCGACCAACGG	pXPR_050	10090	16414	Itgb2	10090	16414	Itgb2	3404108.711
C09	BRDN0007709560	GCCTCCACCCTCCAACAACG	GCCTCCACCCTCCAACAACG	pXPR_050	10090	16822	Lcp2	10090	16822	Lcp2	10579412-maxOut
C10	BRDN0007709559	AAAACCGAGCACCATCAATG	AAAACCGAGCACCATCAATG	pXPR_050	10090	16822	Lcp2	10090	16822	Lcp2	10579412-maxOut
C11	BRDN0007709447	GGCCGGTGCGGCGACTACGG	GGCCGGTGCGGCGACTACGG	pXPR_050	10090	16995	Ltb4r1	10090	16995	Ltb4r1	10579412-maxOut
C12	BRDN0007709327	ACTGTACGGTACACAAGGAC	ACTGTACGGTACACAAGGAC	pXPR_050	10090	16995	Ltb4r1	10090	16995	Ltb4r1	10579412-maxOut
D01	BRDN0007709480	AGAGGAACAAGGTGACATTG	AGAGGAACAAGGTGACATTG	pXPR_050	10090	17096	Lyn	10090	17096	Lyn	10579412-maxOut
D02	BRDN0007709479	CCAACTTAATGGACTCCCGG	CCAACTTAATGGACTCCCGG	pXPR_050	10090	66,651,317,096	Gm11787,Lyn	10090	17096	Lyn	10579412-maxOut
D03	BRDN0007709531	TCGTTGTAAGATCGGTACTG	TCGTTGTAAGATCGGTACTG	pXPR_050	10090	17523	Mpo	10090	17523	Mpo	10579412-maxOut
D04	BRDN0007709407	GGTTATGCTCTCGAACAAAG	GGTTATGCTCTCGAACAAAG	pXPR_050	10090	17523	Mpo	10090	17523	Mpo	10579412-maxOut
D05	BRDN0007709513	GGAACAGCGCATGATAACTG	GGAACAGCGCATGATAACTG	pXPR_050	10090	18795	Plcb1	10090	18795	Plcb1	10579412-maxOut
D06	BRDN0007709332	CAAATACTTACGAATCCACG	CAAATACTTACGAATCCACG	pXPR_050	10090	18795	Plcb1	10090	18795	Plcb1	10579412-maxOut
D07	BRDN0007709536	GGGTGATGTCGACGTCAACG	GGGTGATGTCGACGTCAACG	pXPR_050	10090	234779	Plcg2	10090	234779	Plcg2	10579412-maxOut
D08	BRDN0007709349	GCGGATATACGCTTCCGTGG	GCGGATATACGCTTCCGTGG	pXPR_050	10090	234779	Plcg2	10090	234779	Plcg2	10579412-maxOut

D09	BRDN0007709558	CCGAGTTCAACTCGCTCCCG	CCGAGTTCAACTCGCTCCCG	pXPR_050	10090	378460	Pram1	10090	378460	Pram1	10579412-maxOut
D10	BRDN0007709369	TGGGGGAACTCATAGAACAT	TGGGGGAACTCATAGAACAT	pXPR_050	10090	378460	Pram1	10090	378460	Pram1	10579412-maxOut
D11	BRDN0007709540	CATGGAATACGTCAACGGCG	CATGGAATACGTCAACGGCG	pXPR_050	10090	18750	Prkca	10090	18750	Prkca	10579412-maxOut
D12	BRDN0007709539	GTGGATAAGTCCATAGAGCA	GTGGATAAGTCCATAGAGCA	pXPR_050	10090	18750	Prkca	10090	18750	Prkca	10579412-maxOut
E01	BRDN0007709543	AGTGTTCCCTCAATCCAGAG	AGTGTTCCCTCAATCCAGAG	pXPR_050	10090	18751	Prkcb	10090	18751	Prkcb	10579412-maxOut
E02	BRDN0007709542	ACTGACCACACGGAACGCCG	ACTGACCACACGGAACGCCG	pXPR_050	10090	18751	Prkcb	10090	18751	Prkcb	10579412-maxOut
E03	BRDN0007709541	ATATGGATCTCATCCGACGT	ATATGGATCTCATCCGACGT	pXPR_050	10090	18752	Prkcg	10090	18752	Prkcg	175220.9382
E04	BRDN0007709475	AAGCCACACTGAATCCCGTG	AAGCCACACTGAATCCCGTG	pXPR_050	10090	18752	Prkcg	10090	18752	Prkcg	10579412-maxOut
E05	BRDN0007709561	TCTTTGTCAAATCTGTACAC	TCTTTGTCAAATCTGTACAC	pXPR_050	10090	14083	Ptk2	10090	14083	Ptk2	10579412-maxOut
E06	BRDN0007709499	CAAGCAGCCTACTTACTCGA	CAAGCAGCCTACTTACTCGA	pXPR_050	10090	14083	Ptk2	10090	14083	Ptk2	10579412-maxOut
E07	BRDN0007709372	GCAGTATCCATCAATCAGGT	GCAGTATCCATCAATCAGGT	pXPR_050	10090	14083	Ptk2	10090	14083	Ptk2	10579412-maxOut
E08	BRDN0007709565	GCAGTATGCATCACTCCGGG	GCAGTATGCATCACTCCGGG	pXPR_050	10090	19229	Ptk2b	10090	19229	Ptk2b	10579412-maxOut
E09	BRDN0007709436	ACAGCAACAGCTTCAACCCA	ACAGCAACAGCTTCAACCCA	pXPR_050	10090	19229	Ptk2b	10090	19229	Ptk2b	10579412-maxOut
E10	BRDN0007709375	TTGATAAAAGTACAGCAATG	TTGATAAAAGTACAGCAATG	pXPR_050	10090	19229	Ptk2b	10090	19229	Ptk2b	135840.6198
E11	BRDN0007709470	GTAAGGTCGTGACCGCCATG	GTAAGGTCGTGACCGCCATG	pXPR_050	10090	19303	Pxn	10090	19303	Pxn	194016.0902
E12	BRDN0007709411	TTTCTGGGATGCCATAAAGG	TTTCTGGGATGCCATAAAGG	pXPR_050	10090	19303	Pxn	10090	19303	Pxn	10579412-maxOut
F01	BRDN0007709391	GATCAAGCTTCGTCCCCACG	GATCAAGCTTCGTCCCCACG	pXPR_050	10090	19353	Rac1	10090	19353	Rac1	10579412-maxOut
F02	BRDN0007709390	AGGAGGATACTCACACGGTG	AGGAGGATACTCACACGGTG	pXPR_050	10090	19353	Rac1	10090	19353	Rac1	10579412-maxOut
F03	BRDN0007709517	AAAACACATCAATATAACAT	AAAACACATCAATATAACAT	pXPR_050	10090	11848	Rhoa	10090	11848	Rhoa	10579412-maxOut
F04	BRDN0007709389	TGTAATTGTCAAACACAGTG	TGTAATTGTCAAACACAGTG	pXPR_050	10090	56212	Rhog	10090	56212	Rhog	10579412-maxOut
F05	BRDN0007709551	CTTAACCATTCCCTTCGAAG	CTTAACCATTCCCTTCGAAG	pXPR_050	10090	78473	Skap1	10090	78473	Skap1	137332.2986
F06	BRDN0007709429	AAGGATGCTTCTGACGTGAG	AAGGATGCTTCTGACGTGAG	pXPR_050	10090	78473	Skap1	10090	78473	Skap1	10579412-maxOut
F07	BRDN0007709554	GGTTTGAATGGCAGAAACGG	GGTTTGAATGGCAGAAACGG	pXPR_050	10090	54353	Skap2	10090	54353	Skap2	10579412-maxOut
F08	BRDN0007709489	ATCATCCTCAGGAATAACGT	ATCATCCTCAGGAATAACGT	pXPR_050	10090	54353	Skap2	10090	54353	Skap2	10579412-maxOut
F09	BRDN0007709525	TTACCTCAATGGCTAAACCC	TTACCTCAATGGCTAAACCC	pXPR_050	10090	20619	Snap23	10090	20619	Snap23	10579412-maxOut
F10	BRDN0007709398	AATAAATAAAGACATGAGAG	AATAAATAAAGACATGAGAG	pXPR_050	10090	20619	Snap23	10090	20619	Snap23	10579412-maxOut
F11	BRDN0007709532	TGCAAAACCTCTCAGAACAG	TGCAAAACCTCTCAGAACAG	pXPR_050	10090	20846	Stat1	10090	20846	Stat1	10579412-maxOut
F12	BRDN0007709468	TGTGATGTTAGATAAACAGA	TGTGATGTTAGATAAACAGA	pXPR_050	10090	20846	Stat1	10090	20846	Stat1	10579412-maxOut
G01	BRDN0007709522	ATTACAACTCAGTCAACACA	ATTACAACTCAGTCAACACA	pXPR_050	10090	20909	Stx4a	10090	20909	Stx4a	128978.8977
G02	BRDN0007709461	CGAATACCGAGAGAAGAATG	CGAATACCGAGAGAAGAATG	pXPR_050	10090	20909	Stx4a	10090	20909	Stx4a	10579412-maxOut
G03	BRDN0007709502	GTCTTGGGCTGTACTCCCGG	GTCTTGGGCTGTACTCCCGG	pXPR_050	10090	20963	Syk	10090	20963	Syk	10579412-maxOut
G04	BRDN0007709438	ATTGCACTACCGCATTGACA	ATTGCACTACCGCATTGACA	pXPR_050	10090	20963	Syk	10090	20963	Syk	10579412-maxOut
G05	BRDN0007709521	AAAGATACACAATCCTGTCT	AAAGATACACAATCCTGTCT	pXPR_050	10090	20955	Vamp7	10090	20955	Vamp7	10579412-maxOut
G06	BRDN0007709520	AATAAACTAACTTACTCACA	AATAAACTAACTTACTCACA	pXPR_050	10090	20955	Vamp7	10090	20955	Vamp7	10579412-maxOut
G07	BRDN0007709538	CTTTGAGCGACGCTCCACTG	CTTTGAGCGACGCTCCACTG	pXPR_050	10090	22324	Vav1	10090	22324	Vav1	10579412-maxOut
G08	BRDN0007709537	AACGACGAAGATATTTACAG	AACGACGAAGATATTTACAG	pXPR_050	10090	22324	Vav1	10090	22324	Vav1	9083227.961
G11	BRDN0003227957	GGCCACAAGTTCAGCGTGTC	GGCCACAAGTTCAGCGTGTC	pXPR_050	10090			CONTROL	eGFP	eGFP	10579412-maxOut
H02	BRDN0003228022	GCTGAAGGGCATCGACTTCA	GCTGAAGGGCATCGACTTCA	pXPR_050	10090			CONTROL	eGFP	eGFP	10579412-maxOut
H05	BRDN0000585515	CCCGGCGCCATTCTATCCGC	CCCGGCGCCATTCTATCCGC	pXPR_003	10090			CONTROL (NTC)	Luciferase	Luciferase	10579412-maxOut
H07	BRDN0000585533	ATCAAGGCGTTGGTCGCTTC	ATCAAGGCGTTGGTCGCTTC	pXPR_003	10090			CONTROL (NTC)	Luciferase	Luciferase	10579412-maxOut
H09	BRDN0000562876	GGAGCGCACCATCTTCTTCA	GGAGCGCACCATCTTCTTCA	pXPR_003	10090			CONTROL	eGFP	eGFP	10579412-maxOut
H11	BRDN0001058050	ATCAAGGCGTTGGTCGCTTC	ATCAAGGCGTTGGTCGCTTC	pXPR_003	10090			CONTROL (NTC)	Luciferase	Luciferase	10579412-maxOut
