# Supplementary material for: Neutrophil degranulation and extracellular ROS production are inactivated by Yersinia pseudotuberculosis YopE through a SKAP2 independent pathway
Source: mBio. 2026 Oct 6;17(11):e01984-26. doi: 10.1128/mbio.01984-26 (PMC13753789; doi:10.1128/mbio.01984-26)
Supplement: Supplementary file 2 [file mbio.01984-26-s0008.docx]

**Supplemental Figure Legends:**

**Neutrophil degranulation and extracellular ROS production are inactivated by *Yersinia pseudotuberculosis* YopE through a SKAP2 independent pathway**

Pathricia A. Leus^1^, Claudia Mañan Mejias^1^, Alison Ren^2^, Marjorie de la Rosa^2^, Emma Lofgren^1^, Steve C. Bunnell^3^, David B. Sykes^4^, Joan Mecsas^1,2^

^1^Graduate Program in Molecular Microbiology, Tufts Graduate School of Biomedical Sciences, Boston, USA.

^2^Department of Molecular Biology and Microbiology, Tufts University School of Medicine, Boston, USA.

^3^Department of Immunology, Tufts University School of Medicine, Boston, USA.

^4^Center for Regenerative Medicine, Massachusetts General Hospital, Boston, MA

#Address correspondence to Joan Mecsas, [joan.mecsas@tufts.edu](mailto:joan.mecsas@tufts.edu)

**Supplemental Figure Legends**

**Supplementary Figure 1: Gating Strategy** (A) Debris was gated out using FSC-A v. SSC-A, followed by gating for single cells using FSC-A v. FSC-H. Single Cells were analyzed with a Viability Dye. Viability Dye negative single cells were subsequently analyzed for CD11b and Gr-1 expression. CD11b+Gr-1+ population was further analyzed for percentage of CD63+ or CD63 geometric Mean Fluorescence Intensity (gMFI) as indicated in the figure legend. (B) Representative gating for CD11b+Gr-1+ population plotted in Fig. 1E is shown for CG12-HoxB8 (in grey) and CG12-PMN (in black). (C) Representative dot plots for CD63 plasma membrane localization of FSC-A v. SSC-A, singlets expressing CD11b+Gr-1+ populations are plotted for CG12 uninfected, CG12 infected with *Yptb-*WT, or *Yptb-∆5.* Representative gating for percentage of CD63+ is shown.

**Supplemental Figure 2: YopK is not necessary for blocking CD63 plasma membrane localization** CG12-PMNs were infected with the indicated strains of *Yptb* at an MOI of 30 for 1 hour at 37°C. ∆*yopK* is a strain of *Yptb* lacking YopK. *∆5* is a strain of *Yptb* lacking the 5 Yop effectors: YopE, YopH, YopO, YopJ, and YopM; ∆6 is a strain of *Yptb* lacking 6 Yop effectors: YopE, YopH, YopO, YopJ, YopM, and YopK. After infection, cells were stained, fixed, and analyzed on Aurora Cytek Spectral Cytometer. Gating Strategy is shown in Supplemental Figure 1 (Fig. S1). Percentage of cells with CD63 localized to the plasma membrane are plotted. Statistical significance was calculated using One-Way ANOVA followed by Sidak’s multiple comparison test.

**Supplemental Figure 3: Validating secretion of Yop proteins in Yop-only strains *Yptb* strains** WT, ∆5, YopH-only, YopO-only, and YopE-only were grown in LCM at 26°C for 2 hours and shifted to 37°C for another 2 hours. Yops secreted in the supernatant were precipitated with Trichloroacetic acid (TCA), resolved on 10% polyacrylamide gel, and stained with Coomassie Blue.

**Supplemental Figure 4: Preliminary Screen with pooled knockout cell lines** (A) Histogram of surface localized CD63 on uninfected samples and *Yptb-∆5-*infected samples: CG12-PMNs (black, dashed), two *Cd63*-/- KO-PMN pools generated with two different gRNAs (blue), and unstained CG12-PMN samples (grey, dashed). (B-C) Each well of a 96-well plate containing CG12 were transduced with a gRNA targeting the indicated gene on the x-axis. After 9 days of 2 µg/mL puromycin selection, 2.5-3 x 10^4^ cells per well were seeded into two 96-well plates and differentiated for 3 days. Cells were (B) infected with *Yptb-∆5* at an MOI of 30 or (C) left uninfected for 1 hour, and processed for CD63 plasma membrane expression. Cells were analyzed on BD FACSCelesta. (B) CD63 geometric Mean Fluorescence Intensity (gMFI) of cell lines infected with *Yptb-*∆5; values 1 standard deviation above (green circles) or below (blue circles) are indicated. Shaded regions represent mean ± 1 SD, with mean being marked with a solid line. n=1 (C) Normalized CD63 gMFI relative to parental CG12 (set at 1) for uninfected parental pools of cell lines that were single cell cloned. NTC, non-targeting control using a gRNA targeting luciferase.

**Supplemental Figure 5: Validation of KO in single cell clones using Western Blot** Lysates from differentiated (B-E, I, K) or undifferentiated (A, F-H, J) single cell clones and CG12-PMN were resolved on a Bis-Tris Gel and probed with antibodies for the indicated protein: (A) PLCγ2, (B) SYK, (C) SLP-76, (D) PYK2, (E) LYN, (F) VAV1, (G) MPO, (H) BTK, (I) SKAP2, (J) RhoG, or (K) PXN. Abbreviations above lanes indicate the unique gRNA ID (top) and clone number (below). Confirmed single cell clones used in the screen were denoted with an asterisk (*).

**Supplemental Figure 6: Validation of eGFP-KO and *Cybb-/-* clones** (A) CG12, *Cybb-/-* PMN, and *Cybb-/-* clones from sgRNA Well B2 subclones #1-10 were differentiated into PMNs as previously described and assessed for extracellular ROS production after PMA stimulation. Subclone *Cybb-/-* B2-8 was used for screen*(B) CG12, WT-ER-HoxB8, and GFP-KO-GMP clones from sgRNA Well H9 subclones #1-5 were analyzed on LSRII flow cytometer for GFP expression. Subclone eGFP-KO H9-3 was used for screen*.
